# A Supramolecular Material for Controlling Kiwifruit Bacterial Canker

**DOI:** 10.1002/advs.202414752

**Published:** 2025-05-24

**Authors:** Xile Deng, Qiang Bian, Mingqing Zhou, Le Xie, Jichuan Zhang, Tianqi Liu, Yizhuo Zhang, Li Zhang, Jiaheng Zhang, Lianyang Bai

**Affiliations:** ^1^ Hunan Academy of Agricultural Sciences Changsha 410125 China; ^2^ School of Material Sciences and Engineering Harbin Institute of Technology (Shenzhen) Shenzhen 518055 China; ^3^ National Pesticide Engineering Research Center (Tianjin) College of Chemistry Nankai University Tianjin 300071 China; ^4^ Department of Microbiology College of Life Sciences Nankai University Tianjin 300071 China; ^5^ Department of Applied Chemistry College of Science China Agricultural University Beijing 100193 China; ^6^ Department of Chemistry College of Sciences Northeastern University Shenyang 110819 China; ^7^ Department of Neurology Hunan Academy of Chinese Medicine Affiliated Hospital (Hunan Hospital of Integrated Traditional Chinese and Western Medicine) Changsha 410006 China

**Keywords:** bactericidal activity, kiwifruit bacterial canker (KBC), molecular simulations, nano pesticide, supramolecular nanocarrier

## Abstract

Kiwifruit, a nutritious fruit consumed globally, is affected by kiwifruit bacterial canker (KBC) caused by *Pseudomonas syringae* pv. *actinidiae* (*Psa*), which is a major biotic stress that adversely impacts its cultivation and production. KBC control is still challenging owing to the evolution of resistant populations of *Psa*, the environmental risks associated with copper bactericides, and lack of effective bactericides. Therefore, to develop novel and efficient bactericides against *Psa*, a matrine (MT)‐5‐methylsalicylic acid (5‐OMESA) salt (MOS) is synthesized and its antibacterial activity is analyzed. The newly synthesized compound is more antibacterial against *Psa* than the commercial bactericide thiazole copper (TC). MOS significantly disrupts the membrane structure of *Psa* and penetrates the cells more efficiently. In addition, it has high affinities to the *Psa* FtsZ protein and DNA helicase, which probably contribute to its bactericidal activity. Subsequently, the encapsulation of MOS into a supramolecular nanocarrier hydroxypropyl‐β‐cyclodextrin (HPCD) and the fabrication of a nano formulation (MOS@HPCD) result in superior solubility, penetration, foliar deposition and wettability, sustained release, and prolonged protection against *Psa*. The in vitro and in vivo control efficiencies of MOS@HPCD against *Psa* are markedly enhanced compared to those of MOS. This study proposes a promising supramolecular material to control KBC.

## Graphical abstract

The matrine‐5‐methylsalicylic acid salt (MOS) is reported for its excellent antibacterial activity against *Pseudomonas syringae* pv. *actinidiae* (*Psa*), with advanced cell membrane penetration ability and strong affinities for potential targets. Furthermore, a nanosupramolecular delivery system MOS@hydroxypropyl‐beta‐cyclodextrin (HPCD) with improved deposition, absorption, conductivity, transport, and controlled‐release ability is developed. It shows better antibacterial activity against *Psa* than that of MOS.

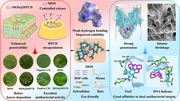

## 1 Introduction

Bacterial diseases pose significant risks to plant growth and development. In fruit crops worldwide, these diseases are known to decrease their quality and yield.^[^ ^1^ ^]^ For instance, in kiwifruit, an economically nutritious fresh fruit consumed globally with an annual production of 4.54 million tons,^[^ ^2^ ^]^ kiwifruit bacterial canker (KBC) caused by *Pseudomonas syringae* pv. *actinidiae* (*Psa*) leads to stem lesions, leaf spots, and flower rot.^[^ ^3^ ^]^ From 2012 to 2021, the economic losses owing to *Psa* in New Zealand, the major country that produces kiwifruit, were estimated to be between 330 and 400 million euros.^[^ ^4^ ^]^ Although various methods are adopted to manage KBC, their implementation remains challenging. Moreover, the continuously evolving resistant populations of *Psa*, the environmental risks associated with the application of copper fungicides, and the lack of effective bactericides against *Psa* limit its management.^[^ ^5^ ^]^

Natural products and their derivatives, including the commercially available natural bactericide‐like matrine (MT), are preferred for controlling plant‐pathogenic bacteria owing to the growing demand for environmental protection.^[^ ^6^ ^]^ MT is a tetracyclic quinoline alkaloid first detected in the Chinese herb *Sophora flavescens*. It has broad‐spectrum antibacterial activity and is effective against *Pseudomonas aeruginosa*, *Staphylococcus epidermidis*, *Staphylococcus aureus*, *Streptococcus mutans*, and *Streptococcus agalactiae*.^[^ ^7^ ^]^ However, the poor solubility and permeability of MT limit its biological activity, which restricts its application in agriculture.^[^ ^8^ ^]^ The solubility and permeability of natural agrochemicals are substantially higher in their salt forms.^[^ ^9^ ^]^ For example, the solubility and permeability of salicylic salts are remarkably higher than those of salicylic acid (SA), and these properties play important roles in pharmaceuticals and agrochemicals.^[^ ^10^ ^]^ For instance, substituted SA salts were highly effective against *Escherichia coli* and *Staphylococcus aureus*.^[^ ^11^, ^12^ ^]^

Various studies have demonstrated that agrochemicals in their salt forms exhibit enhanced properties, including improved solubility and bioavailability.^[^ ^9^ ^]^ However, the salt form of MT has never been reported as a bactericide in agriculture. Therefore, this study aimed to develop a novel, efficient, and eco‐friendly alternative for controlling KBC. To achieve this, we synthesized a new organic ionic salt, MT‐5‐OMESA (MOS), using MT as the cation and 5‐methylsalicylic acid (5‐OMESA) as the anion.^[^ ^12^ ^]^ The bactericidal activity of MOS against *Psa* was evaluated in vitro by determining its inhibition rate and median effect concentration (EC_50_) in comparison to the copper‐based bactericide thiazole copper (TC). To elucidate the molecular basis of MOS's antibacterial activity, we investigated its ability to penetrate the bacterial cell membrane and interact with key bacterial targets, such as the FtsZ protein and DNA helicase, through molecular dynamics (MD) simulations.^[^ ^3^, ^13^ ^]^ Since the solubility and delivery efficiency of MOS in aqueous environments could affect its practical application, we further developed a supramolecular nano‐delivery system (MOS@HPCD) using hydroxypropyl‐beta‐cyclodextrin (HPCD) as a carrier, enabling sustained release and enhanced bioavailability. Finally, the toxicity tests of MOS and MOS@HPCD on toxicological models, such as zebrafish (*Danio rerio*) embryos, mice, and rats, were assessed to ensure their environmental safety. This study demonstrates that MOS exerts its antibacterial activity against *Psa* through multiple biological mechanisms. First, MOS destabilizes the bacterial cell membrane, increasing permeability and inducing physiological stress. Second, MOS directly targets essential bacterial proteins, FtsZ and DNA helicase, which are critical for bacterial cell division and replication. Computational simulations further confirm its strong binding affinities and ability to disrupt bacterial function. Finally, the supramolecular formulation MOS@HPCD enhances MOS's solubility, absorption, and adhesion to plant surfaces, ensuring prolonged antibacterial action. These findings provide a new approach to designing agrochemicals with enhanced efficacy, offering an innovative and eco‐friendly solution for controlling KBC. By integrating material synthesis, antibacterial evaluation, molecular simulation, and plant application studies, this work establishes MOS as a promising candidate for sustainable plant disease management.

## 2 Results and Discussion

### 2.1 Synthesis and Characterization of MOS

**Figure 1:**
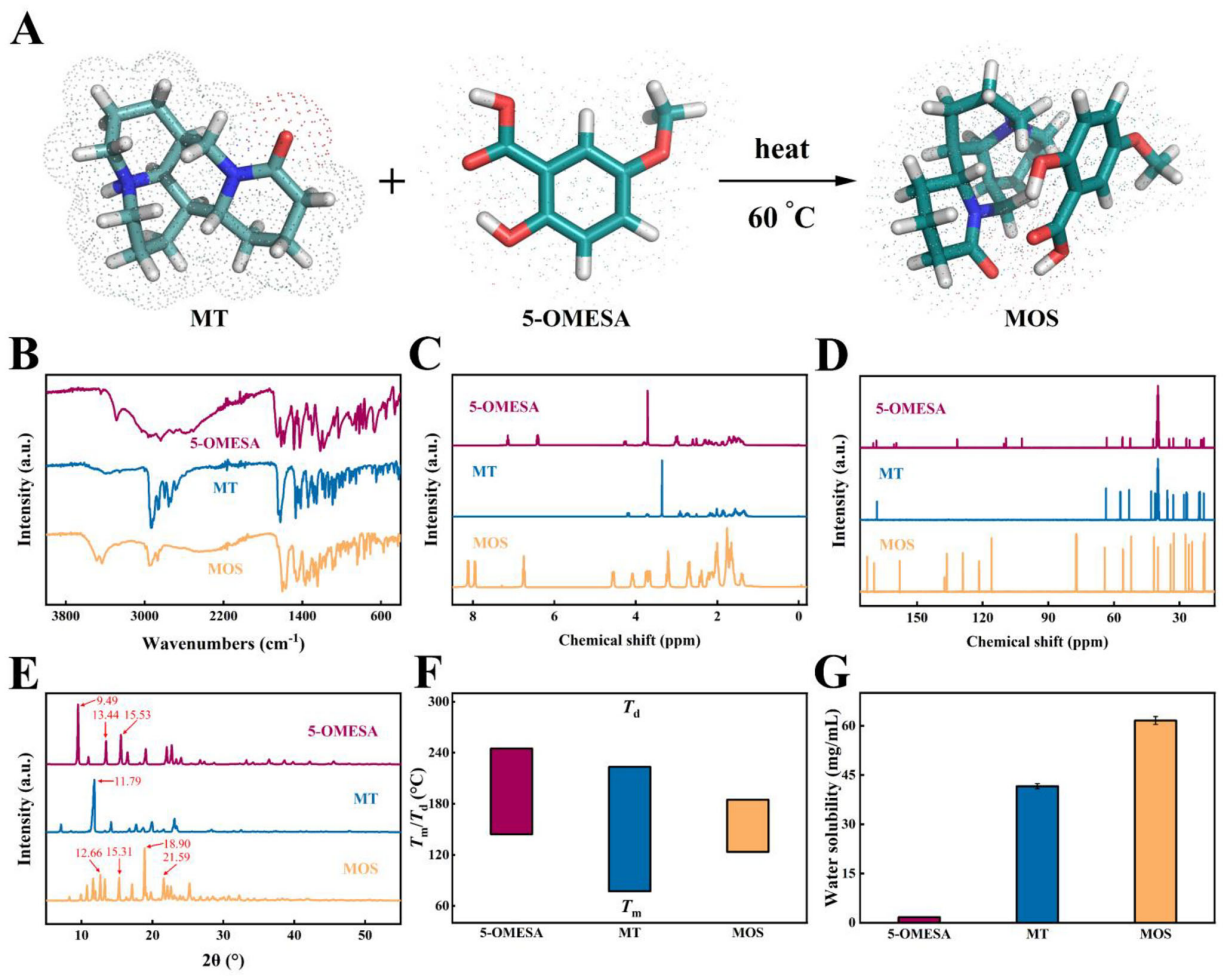
Synthesis and characterization of MOS. A) Synthesis route of MOS. The white, lake blue, red, and blue sticks represent hydrogen, carbon, oxygen, and nitrogen atoms, respectively. B) IR spectra of 5‐OMESA, MT, and MOS. C) ^1^H NMR spectra of 5‐OMESA, MT, and MOS. D) ^13^C NMR spectra of 5‐OMESA, MT, and MOS. E) XRD patterns of 5‐OMESA, MT, and MOS. F) Onset *T*_m_ (down)/*T*_d_ (up) values of 5‐OMESA, MT, and MOS. G) Water solubilities of 5‐OMESA, MT, and MOS (*n* = 3). The results are shown as the mean ± SD. Red: 5‐OMESA; blue: MT; yellow: MOS.

MOS was synthesized by an atom‐economic quaternization reaction using the commercially available reagents MT and 5‐OMESA (**Figure** **1**A). First, 5‐OMESA and MT were heated for 1 h in methanol and left to form a colorless MOS crystal with a yield of 90.2 wt.% at room temperature. A single crystal of MOS with a size of 0.15 × 0.10 × 0.08 mm^3^ was selected, and its structure was assessed using single‐crystal X‐ray diffraction (XRD) at 169.99(10) K. The analysis revealed that the MOS is a monoclinic crystal system (the P2_1_2_1_2_1_ space group) with a density of 1.312 g cm⁻^3^. In addition, it had the following cell parameters: a = 9.5156(2) Å; b = 13.4691(4) Å; c = 17.1637(5) Å; α = 90°; β = 90°; γ = 90°; Z = 4; and Volume = 2199.81(10) Å3 (Table S1, Supporting Information).

The structure and composition of MOS were analyzed further using infrared radiation (IR), nuclear magnetic resonance (NMR), and X‐ray powder diffractometer (XRD) spectroscopy. The IR spectrum of 5‐OMESA displayed a wide O‐H stretching band, ranging from 2489 to 3042 cm^−1^, which corresponded to the overlay of phenolic hydroxyl and carboxyl groups.^[^ ^14^ ^]^ The peak that corresponded to the C═O bond stretching was observed around 1648 cm^−1^.^[^ ^15^ ^]^ These characteristic peaks also appeared in the IR spectrum of MOS (Figure 1B). Additionally, a broad N^+^‐H stretching peak was detected around 2466 cm^−1^, and the symmetric and anti‐symmetric peaks of carboxylate anion were observed at 1614 cm^−1^ and 1421 cm^−1^, respectively, in the IR spectrum of MOS.^[^ ^16^ ^]^ The shift in the O─H stretching peak to the 3421–3486 cm^−1^ range revealed that 5‐OMESA lost the proton hydrogen during the reaction.^[^ ^17^ ^]^ Furthermore, the ^1^H NMR and ^13^C NMR spectra of 5‐OMESA, MT, and MOS were analyzed (Figure 1C,D). The peaks attributed to the protons on the phenyl ring appeared at 6.3 and 7.1 ppm for 5‐OMESA, but a proton loss on the carboxyl group in 5‐OMESA resulted in a shift to a higher field. In the MOS spectrum, these peaks appeared around 6.7 and 7.9 ppm (Figure 1C). The peak owing to the carbon atom in the C═O bond appeared at 170 ppm in 5‐OMESA, while it shifted to 173 ppm in MOS. This shift suggests the occurrence of quaternization between 5‐OMESA and MT (Figure 1D).^[^ ^18^ ^]^ Furthermore, the PXRD pattern of MOS had characteristic peaks at 12.66, 15.31, 18.90, and 21.59°, which differed from those of 5‐OMESA and MT. The findings indicated the successful synthesis of MOS. (Figure 1E). Moreover, the melting temperatures (*T*_m_) of 5‐OMESA, MT, and MOS were lower than their decomposition temperatures (*T*_d_), which indicated that decomposition did not occur before melting. Notably, the *T*_d_ of MOS (184.83 °C) was much lower than that of 5‐OMESA (244.9 °C) and MT (223.3 °C) (Figure 1F). Moreover, MOS (61.6 g L⁻^1^) was 36.2‐fold more soluble in water than 5‐OMESA (1.7 g L⁻^1^) and 1.48‐fold higher than that of MT (41.6 g L⁻^1^) (Figure 1G). The elemental analysis also indicated a quaternization reaction between MT and 5‐OMESA and that MOS was highly pure (Table S2, Supporting Information).

**Figure 2:**
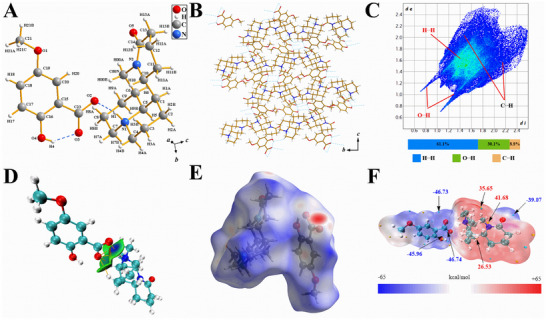
A) Crystal structure of MOS. The gray, white, red, and blue balls represent carbon, hydrogen, oxygen, and nitrogen atoms, respectively. The yellow sticks represent chemical bonds. The blue dotted lines represent hydrogen bond interactions. B) 3‐D layer‐by‐layer stacking with hydrogen bonds viewed along the a‐axis. The lake blue dotted lines represent hydrogen bonding interactions. C) 2‐D fingerprint plot and atom‐atom contact contributions of MOS. D) IGHM isosurfaces of MOS in water. Green region, isosurface; blue region, strong hydrogen bonding interaction. The blue‐green, white, red, and blue balls represent carbon, hydrogen, oxygen, and nitrogen atoms, respectively. E) 3‐D Hirshfeld d_norm_ surface of MOS. The black, red, and lake blue balls represent carbon, oxygen, and nitrogen atoms, respectively, and the white sticks represent hydrogen atoms; Red spots correspond to weak intermolecular interactions. F) Electrostatic potential (ESP) of MOS (red: positively charged; blue: negatively charged).

A subsequent analysis showed that the single X‐ray structure of MOS consisted of a 5‐OMESA anion and an MT cation (**Figure** **2**A) after the molecule formed ionic salts through ionic bonds, and the protons were transferred from carboxylic acid to pyridine N. These reactions formed additional intermolecular and intramolecular hydrogen bonds. These bonds included O4‐H4⋅⋅⋅O3 (2.535 Å) and N1‐H1⋅⋅⋅O2 (2.740 Å). The interaction of ionic and hydrogen bonds probably enhanced the stability of MOS (Table S3, Supporting Information). In addition, the two molecular layers were found to be arranged alternately along the c‐axis; they had a dihedral angle of 76.414° between the phenyl rings of the anions and were connected through N1‐H1⋅⋅⋅O2 hydrogen bonds (Figure 2B). The two‐dimensional (2‐D) fingerprint plot of MOS showed prominent H⋅⋅⋅H, O⋅⋅⋅H, and C⋅⋅⋅H contacts, which accounted for 34.5%, 37.7%, and 14.9%, respectively (Figure 2C). The O⋅⋅⋅H weak interactions in the MOS were distributed symmetrically as intermolecular (*de*) and intramolecular (*di*) interactions. In 5‐OMESA, the total O⋅⋅⋅H contacts (37.7%) in MOS were higher than those in 5‐OMESA (30.1%). Additionally, the intermolecular interaction distance (*de*) in MOS was ≈0.85, while that (*di*) in 5‐OMESA was ≈1.0. These differences suggest that the intermolecular interaction in MOS was stronger than that in 5‐OMESA (Figure S1, Supporting Information). Consistent with this observation, the intermolecular interaction in MOS was also stronger than that of MT (Figure S2, Supporting Information). Generally, a stronger O⋅⋅⋅H intermolecular interaction indicates that the compound is more soluble in an aqueous solution. Thus, the stronger intermolecular interaction in MOS indicates stronger solubility for MOS than for 5‐OMESA and MT. The solubility test proved this hypothesis (Figure 1G). Moreover, the independent gradient model based on Hirshfeld partitioning (IGMH) used to study the interactions visually displayed a blue region in the isosurface, which corroborated the existence of robust hydrogen bonding interactions between [MT]^+^ and [5‐OMESA]^−^ (Figure 2D). The three‐dimensional (3‐D) dnorm surfaces of MOS confirmed the presence of strong intramolecular and weak intermolecular hydrogen bonding interactions (Figure 2E). Additionally, [MT]^+^ showed the most positive electrostatic potential (ESP) value at nitrogen (N2) (41.68 kcal mol⁻^1^), and [5‐OMESA]^−^ displayed the largest negative ESP value at oxygen (O2) (−46.74 kcal mol⁻^1^). These ESP values indicate that both [MT]^+^ and [5‐OMESA]^−^ are highly polar, which enhances their solubility in water (Figure 2F). The changes in the IR spectra of 5‐OMESA after the formation of salt with MT also suggest that there was weaker hydrogen bonding between the MOS molecules, which proved that they were more soluble in water (Figure 1C). These results demonstrated a negative charge on [5‐OMESA]^−^, and both negative and positive charges over [MT]^+^ in water support the enhanced solubility of MOS in water. The hydrogen bonding interactions between [MT]^+^ and [5‐OMESA]^−^ further proved this feature.

### 2.2 In Vitro Antibacterial Assay of MOS

**Figure 3:**
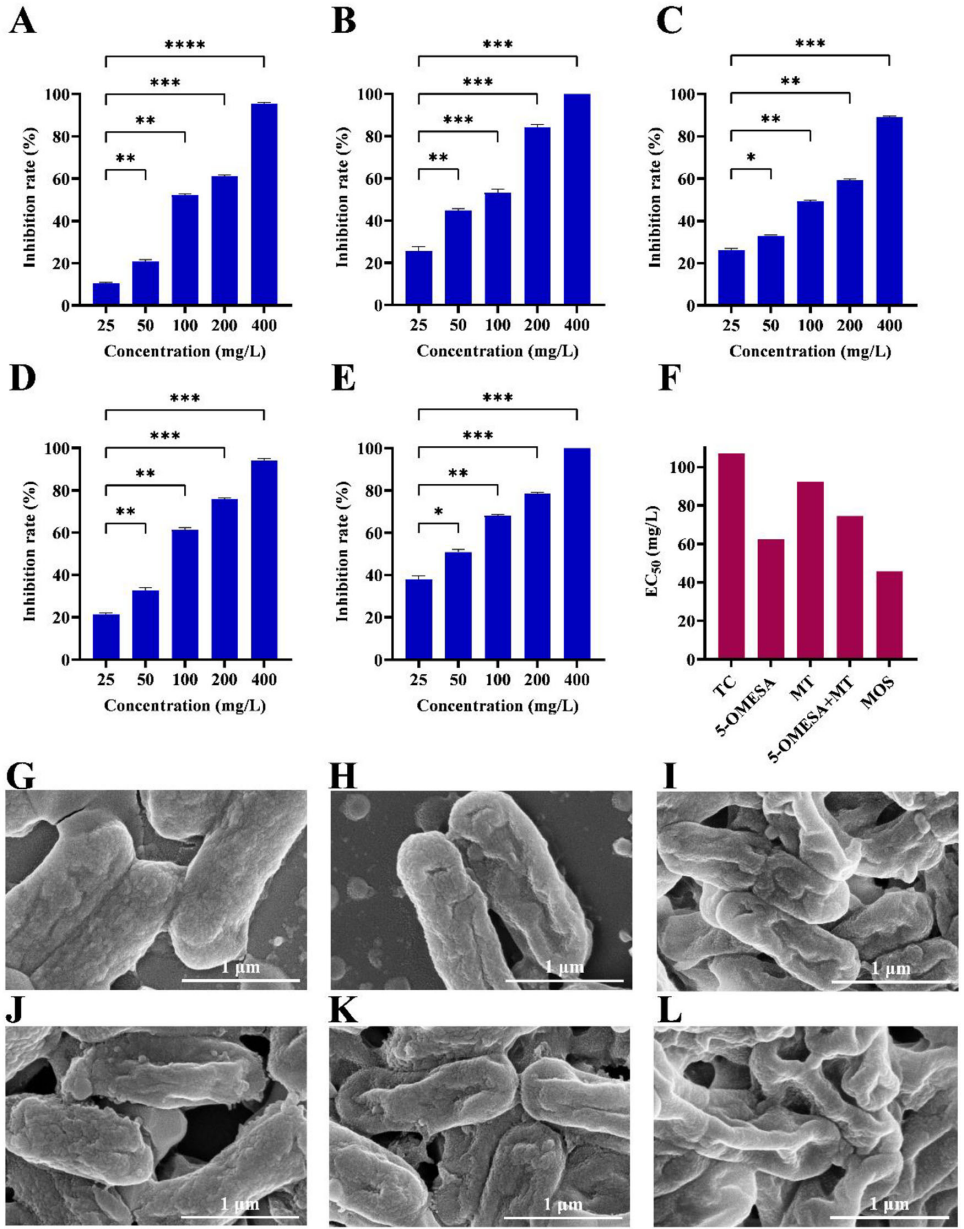
In vitro antibacterial assay of A) TC, B) 5‐OMESA, C) MT, D) 5‐OMESA+MT (molar ratio of 1:1), and E) MOS against *Psa* at 25, 50, 100, 200, and 400 mg L⁻^1^ after 48 h. *n* = 3. Results are shown as mean ± standard deviation. The statistical analysis was performed with ANOVA analysis. *p < 0.05, **p < 0.01, ***p < 0.001, ****p < 0.0001. F) In vitro EC_50_ values of TC, 5‐OMESA, MT, 5‐OMESA+MT (molar ratio of 1:1), and MOS against *Psa* after 48 h. EC_50_ values were determined by probit regression modeling, using inhibition rates from three replicates at 25, 50, 100, 200, and 400 mg L⁻^1^. SEM images of the *Psa* cells treated with G) CK, H) TC, I) 5‐OMESA, J) MT, K) 5‐OMESA+MT (molar ratio of 1:1), and L) MOS at 200 mg L⁻^1^ for 4 h. Scale bar: 1 µm. *Psa*, *Pseudomonas syringae* pv. *actinidiae*.

The in vitro antibacterial activities of the commercial bactericide TC, 5‐OMESA, MT, 5‐OMESA+MT (molar ratio of 1:1), and MOS against *Psa* were evaluated at concentrations that ranged from 25 to 400 mg L⁻^1^ (**Figure** **3**A–F). Notably, MOS had the most effective inhibition (67.9%) at 100 mg L⁻^1^, followed by 5‐OMESA+MT (60.3%), 5‐OMESA (53.3%), TC (52.1%), and MT (49.4%). With the decrease in concentration from 100 to 25 mg L⁻^1^, the trend observed in the inhibition rate varied as follows: MOS > 5‐OMESA, MT, and 5‐OMESA+MT > TC. Moreover, the concentration that resulted in 50% of the EC_50_ of MOS against *Psa* was 45.83 mg L⁻^1^, which was 1.33‐, 0.36‐, 1.02‐, and 0.63‐fold lower than that of TC (106.99 mg L⁻^1^), 5‐OMESA (62.35 mg L⁻^1^), MT (92.52 mg L⁻^1^), and 5‐OMESA+MT (74.55 mg L⁻^1^), respectively (Figure 3F). These results indicated that after ionization, the in vitro antibacterial activity of MOS against *Psa* increased dramatically compared to those of 5‐OMESA and MT. The enhanced antibacterial efficacy of MOS compared to the 5‐OMESA+MT combination was primarily attributed to the ionization of MOS. Moreover, the lowest concentration of MOS (25 mg L⁻^1^) also exhibited higher in vitro antibacterial activity against *Psa* than TC, thus indicating that it could be a prime candidate to control KBC. Although there are a series of nanocarrier‐based bactericides, such as silver‐chitosan copper‐based, organic‐inorganic hybrid, chitosan‐based, and gallic acid‐based nanoparticles,^[^ ^19^ ^]^ with excellent antibacterial activity against *Psa*, MOS has a simple structure and can be more easily synthesized than these reported nanobactericides.

Furthermore, scanning electron microscopy (SEM) was conducted to assess the impact of TC, 5‐OMESA, MT, 5‐OMESA+MT, and MOS on the *Psa* cell membrane using untreated *Psa* with a complete and plump rod‐shaped morphology as the control (Figure 3G–L). The approach showed a negligible effect of MT on the *Psa* cells. Moreover, 5‐OMESA induced the *Psa* cell membrane to wrinkle. However, MOS demonstrated the most pronounced impact on *Psa*. The *Psa* treated with MOS had damage on most of its surfaces and non‐intact cell morphs. SEM analysis (Figure 3G–L) confirmed that MOS treatment led to severe morphological alterations in *Psa*, including cell membrane wrinkling, damage, and loss of structural integrity. These findings suggest that MOS disrupts bacterial biofilm permeability, leading to intracellular solute leakage and physiological stress, ultimately resulting in bacterial cell death. Scientists have shown that salinity damages bacterial cell membranes due to osmotic stress. Hence, our observations suggest that MOS might induce osmotic imbalances, further enhancing membrane damage and bacterial analysis. In addition, the electrostatic attraction between the organic salts and bacterial cell membrane, which results in the disruption of the membrane and the subsequent release of many constituents, serves as a primary factor that contributes to the antibacterial effect of organic salts.^[^ ^20^ ^]^

### 2.3 Bacterial Cell Membrane Penetration

**Figure 4:**
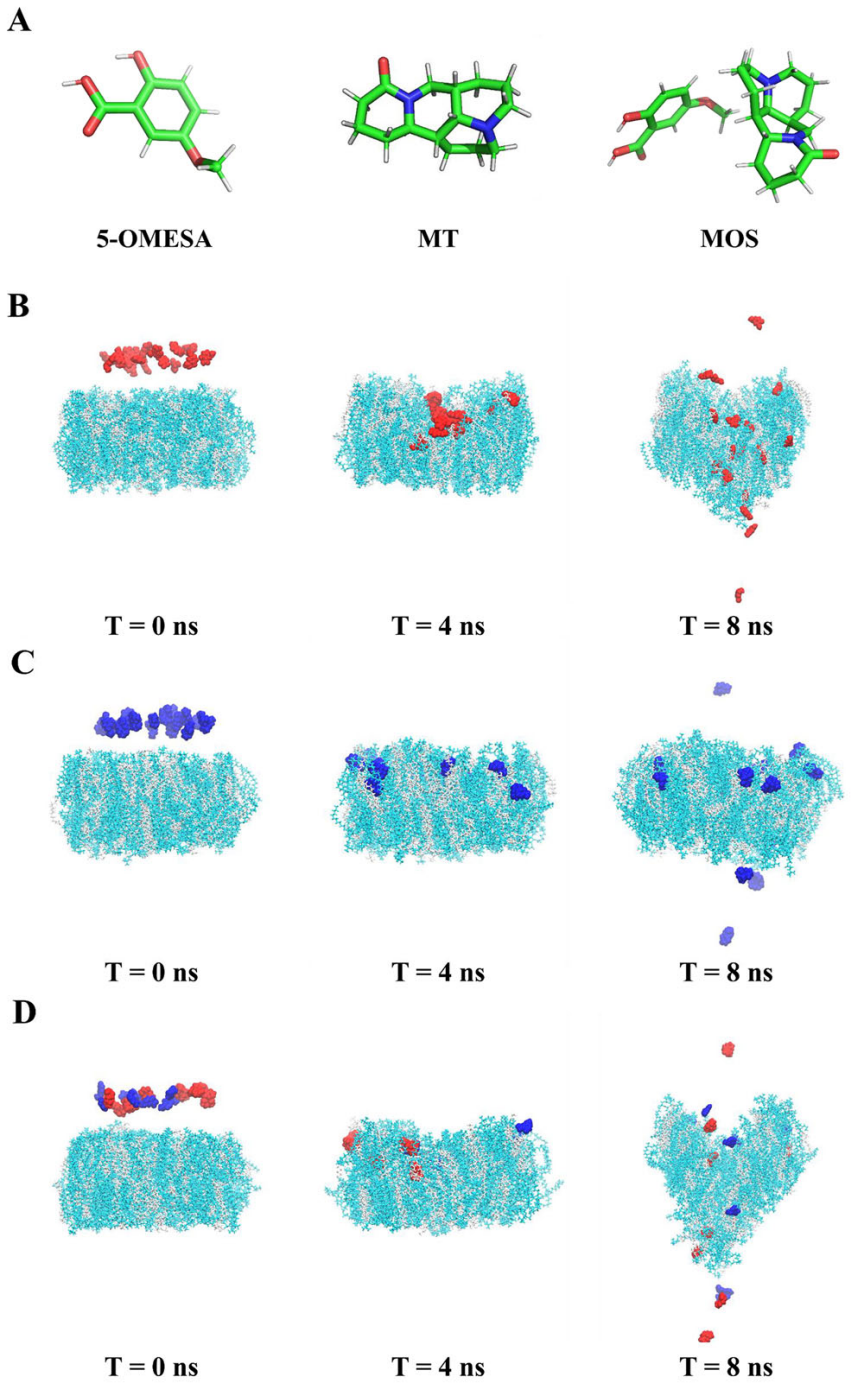
A) Optimized structures of 5‐OMESA, MT, and MOS after the all‐atom MD simulations. The white, green, red, and blue sticks represent hydrogen, carbon, oxygen, and nitrogen atoms. Simulation snapshots jurying the penetration of B) 5‐OMESA (red), C) MT (blue), and D) MOS (red: 5‐OMESA anion; blue: MT cation) into the *Psa* cell membrane at 0, 4, and 8 ns, respectively. *Psa*, *Pseudomonas syringae* pv. *actinidiae*.

**Figure 5:**
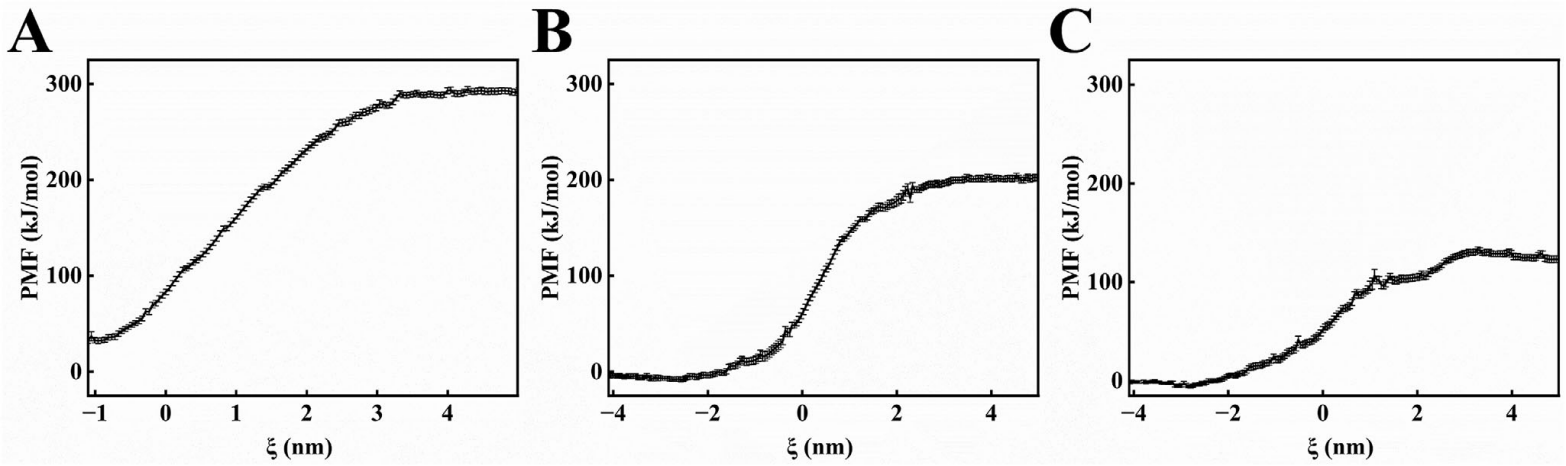
The potential of mean force (PMF) curves of A) 5‐OMESA, B) MT, and C) MOS. *n* = 3. The results are shown as mean ± SD.

The disruption and permeability of the bacterial cell membrane is a crucial indicator of their efficacy. Generally, the targets of bactericides, such as the FtsZ protein and DNA helicase, are within the cells. Therefore, the bactericides must first traverse the cell membrane to interact with their associated targets. Thus, this study conducted molecular simulations to explore the transmembrane mechanisms of 5‐OMESA, MT, and the ionic salt MOS. First, the molecular structure and the force field were generated for 5‐OMESA, MT, and MOS using the CHARMM‐GUI Ligand Reader and Modeler. **Figure** **4**A–D displays the transmembrane processes of 5‐OMESA, MT, and MOS across the bacterial membrane over time (0, 4, and 8 ns). Initially, at 0 ns, all the molecules were located outside the membrane. These molecules partially penetrated the membrane by 4 ns, and most fully penetrated the membrane by 8 ns. This resulted in a significant disruption of the membrane structure. MD trajectories of the membrane penetration of 5‐OMESA, MT, and MOS are shown in Videos S1–S3 (Supporting Information). Furthermore, the free energy barriers surmounted by 5‐OMESA, MT, and MOS to penetrate the *Psa* cell membranes were calculated and compared to assess their differences (**Figure** **5**A–C). The potential of mean force (PMF) curves was employed to estimate the energy barriers associated with the penetration of molecules through the cell membranes. In this study, MOS displayed the smallest ⊿G, which represented the difference between the maximum and minimum free energy values in the PMF curve. These results indicated that the 5‐OMESA and MT molecules interacted to form an ion salt structure (MOS). The MOS synergistically affected the disruptive impact on the cell membrane, thereby reducing the energy barrier. Consequently, MOS penetrated the bacterial cell membrane more easily.

The area per lipid, lipid ordering parameters, and membrane thickness were examined to better understand the disruptive effects on the cell membrane by analyzing the ordering of lipid tails (Sz) (Figure S3, Supporting Information). Both atom tails exhibited S values closer to 0 in all four systems, which indicated a significant disorder. The MOS system displayed the most pronounced fluctuation in the mean surface area occupied by the lipid molecules and membrane thickness compared to 5‐OMESA and MT (Figures S4 and S5, Supporting Information). This fluctuation indicated that the membrane of *Psa* cells was disrupted most significantly. The pronounced fluctuation was particularly obvious in the latter phase of the simulation where a notable reduction in surface area was observed in the MOS system. This reduction in surface area suggests that the cell membrane undergoes substantial structural changes and increase in fluidity during its penetration by MOS. In this study, the action of MOS resulted in the most pronounced disruption in membrane structure compared to 5‐OMESA and MT, which was consistent with the SEM observations on the *Psa* cells (Figure 3G–K). A previous study demonstrated that significant morphological changes in bacterial cell membranes were associated with the antibacterial activities of phenylene ethynylene backbone‐based cationic bactericides.^[^ ^21^ ^]^

Furthermore, the relative distance between each molecule (5‐OMESA, MT, or MOS) and the center of the *Psa* cell membrane was calculated (Figure S6, Supporting Information). The fluctuation in this distance observed for MOS was significantly greater than those for 5‐OMESA and MT, which indicated that MOS was efficiently transported into the *Psa* cells. Thus, the simulation results and the antibacterial activity data revealed that MOS was the most disruptive to *Psa* cell membrane, penetrated the most, and was the most effective at transporting the molecules into the bacterial cells, thus resulting in enhanced antibacterial activity against *Psa*.

### 2.4 Molecular Docking

**Figure 6:**
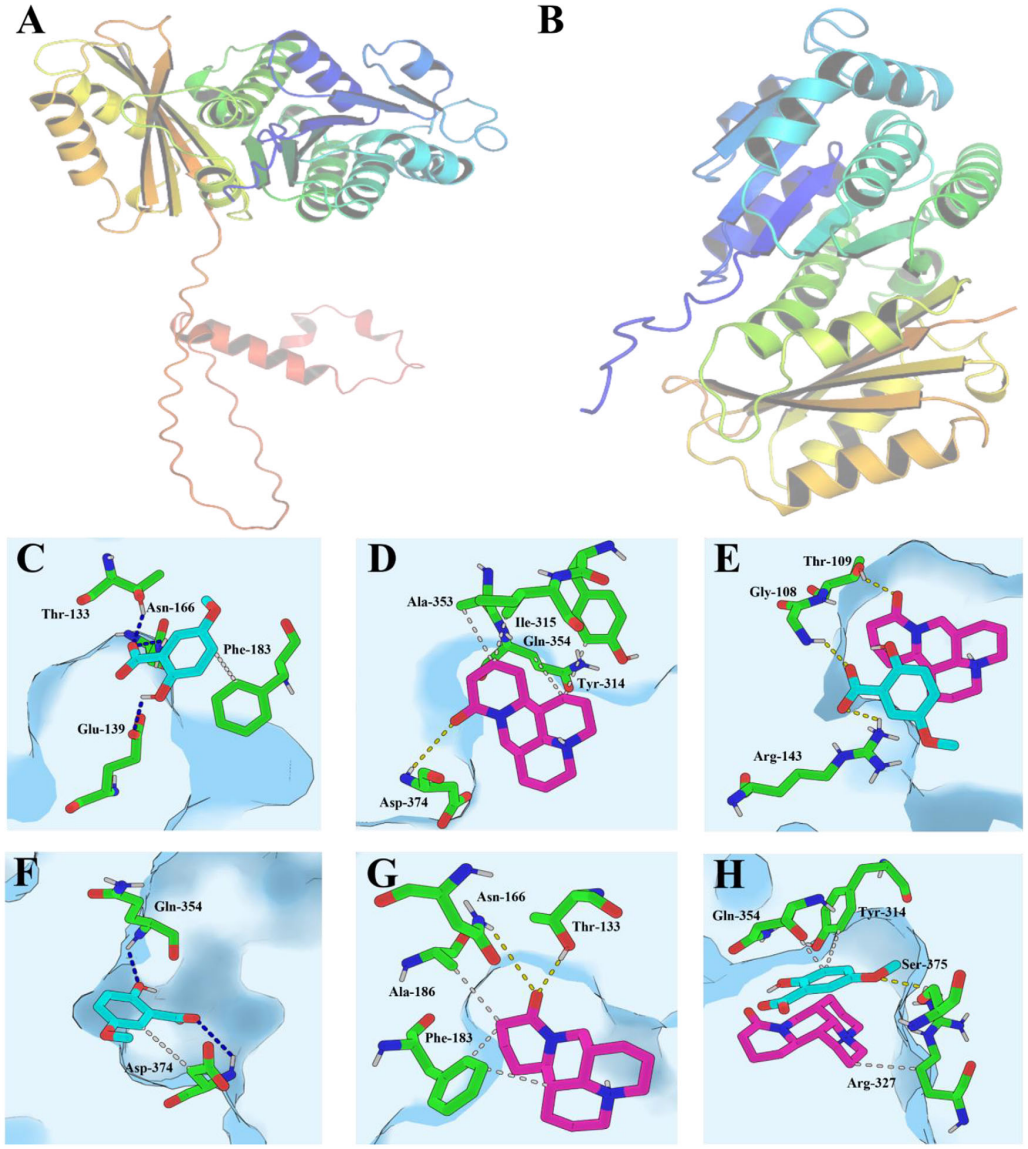
Homologous modeling of A) FtsZ and B) DNA helicase in *Psa* by AlphaFold 3. Interactions between the binding pockets in the targets FtsZ and DNA helicase subunits and the ligands 5‐OMESA (C: FtsZ, F: DNA helicase), MT (D: FtsZ, G: DNA helicase), and MOS (E: FtsZ, H: DNA helicase). The skeletons of 5‐OMESA and MT are colored as lake blue and pink, respectively. Oxygen, red stick; nitrogen; dark blue stick. Hydrogen bonding and hydrophobic interactions as blue or white dotted lines, respectively.

Once compounds, such as 5‐OMESA, MT, and MOS, penetrate the *Psa* cell membrane and enter the cell, they might interact with relevant protein targets to inhibit the normal physiological and metabolic functions of the bacterium. The molecular docking method is widely used to identify small molecules that have a high affinity toward bacterial receptors and explore their action. In this study, we selected FtsZ and DNA helicase, two key bacterial targets, and used AlphaFold 3 to predict their 3D structures (**Figure** **6**A,B).^[^ ^22^ ^]^ Molecular docking simulations were then performed to evaluate the interaction of 5‐OMESA, MT, and MOS with these targets. MOS exhibited the highest binding affinity to both proteins, with docking energies of −6.692 kcal mol⁻^1^ for FtsZ and −6.769 kcal mol⁻^1^ for DNA helicase, significantly lower than those of 5‐OMESA and MT (Table S4, Supporting Information). Previous research suggests that compounds with lower docking energies tend to exhibit stronger bactericidal activity.^[^ ^22^ ^]^ Therefore, these findings suggest that MOS has superior antibacterial potential compared to 5‐OMESA and MT. In the case of FtsZ, 5‐OMESA formed multiple hydrogen bonds with Thr133, Asn166, and Glu139, along with hydrophobic interactions involving Phe183. MT interacted with Asp374 and engaged in several hydrophobic interactions with Tyr314, Ile315, Ala353, and Gln354 (Figure 6C,D). In the case of DNA helicase, 5‐OMESA formed hydrogen bonds with Gln354 and Asp374 and exhibited hydrophobic interactions with Asp374. The top‐ranked conformation of MT included hydrogen bonds with Tyr133 and Asn166 and hydrophobic interactions with Ala186 and Phe183 (Figure 6F,G). In contrast, MOS displayed unique binding profiles, where [MT]+ exhibited hydrophobic interactions with Arg327, while [5‐OMESA]^−^ formed hydrogen bonds with Ser375 and interacted hydrophobically with Tyr314 and Gln354 (Figure 6H). These findings align with previous studies that demonstrated the impact of salt formulations on ligand binding characteristics.^[^ ^22^ ^]^ Overall, these results provide valuable insights into the intermolecular interactions between ligands and bacterial targets, which could aid in the development of new molecular salts to inhibit the activities of FtsZ and DNA helicase in *Psa*. Subsequent experiments pertaining to these targets, such as the GTP activity and assembly of FtsZ on microtubule networks affected by MOS, will be conducted in future research endeavors.

### 2.5 Molecular Simulation

Furthermore, to evaluate the dynamic behavior and simulation stability of 5‐OMESA, MT, and MOS docked into the active sites of *Psa* FtsZ and DNA helicase, MD simulations were conducted for six protein‐ligand complexes, namely FtsZ‐5‐OMESA, FtsZ‐MT, FtsZ‐MOS, DNA helicase‐5‐OMESA, DNA helicase‐MT, and DNA helicase‐MOS. Three MD simulations were conducted for each system, and each one took 10 ns. The interactions were evaluated by quantifying the number of hydrogen bonds generated during the entire molecular simulation process (Figures S7A and S7B, Supporting Information). Beyond molecular docking, MD simulations provided further insights into the stability and dynamic behavior of MOS binding to FtsZ and DNA helicase. The MOS‐FtsZ and MOS‐DNA helicase complexes exhibited a greater number of hydrogen bonds (2.86 for FtsZ‐MOS and 1.79 for DNA helicase‐MOS) compared with the other complexes, further stabilizing their interactions. Generally, ligand binding at the active site leads to a conformational change in protein structure, which can be evaluated using the RMSD value. This study that was based on this approach showed that the RMSDs of FtsZ‐5‐OMESA and FtsZ‐MOS were smaller than that of FtsZ‐MT (Figure S8A, Supporting Information) and were similar to the ones observed in the DNA helicase system (Figure S8B, Supporting Information). This study also showed that the 5‐OMESA and MOS were more stable during the MD simulation and had less conformational variability than MT in the FtsZ and DNA helicase complexes.

The root mean square fluctuation (RMSF) was then calculated to examine the behavior of amino acid residues in FtsZ and DNA helicase after ligand binding.^[^ ^23^ ^]^ The 5‐OMESA and MOS complexes that bound to FtsZ and DNA helicase were larger than those of the MT‐FtsZ and MT‐DNA helicase complexes, which indicated that there were greater fluctuations in the amino acid residues (Figures S9A and S9B, Supporting Information). Furthermore, the radius of gyration (Rg), which measures the structural compactness and overall shape of the protein, was measured.^[^ ^24^ ^]^ The average Rg values of FtsZ‐5‐OMESA, FtsZ‐MT, FtsZ‐MOS, DNA helicase‐5‐OMESA, DNA helicase‐MT, and DNA helicase‐MOS were 2.00, 2.01, 2.01, 2.85, 2.93, and 2.85 nm, respectively, indicating that MOS binding does not significantly perturb the structural integrity of FtsZ (Figures S10A and S10B, Supporting Information). The Rg values of FtsZ‐5‐OMESA and FtsZ‐MOS were smaller than that of FtsZ‐MT, suggesting a more compact and stable conformation in the MD simulation (Figure S10A, Supporting Information). Notably, all Rg values remained relatively stable throughout the MD simulation. Furthermore, FtsZ‐5‐OMESA and FtsZ‐MOS complexes exhibited greater stability than those involving DNA helicase, reinforcing the structural integrity of FtsZ upon MOS binding.

Further, the free energy landscape (FEL) from the MD simulations was visualized and analyzed to obtain insights into the conformational behavior that underlie the protein‐ligand interaction (Figure S11A–F, Supporting Information).^[^ ^25^ ^]^ The shape and size of the minimum energy region indicated the conformational stability of the protein. A wider and deeper dark blue field in the FEL suggested a more stable conformation with lower energy, whereas a dispersed dark region represented greater conformational flexibility of the protein‐ligand complex. As shown in Figure S11E (Supporting Information), the dark field that effectively represents the minimum energy well in FtsZ‐MOS was wider than those in FtsZ‐5‐OMESA (Figure S11A, Supporting Information) and FtsZ‐MT (Figure S11C, Supporting Information), suggesting greater stability. Similarly, the minimum energy well of DNA helicase‐MOS (Figure S11F, Supporting Information) was comparable to that of the DNA helicase‐5‐OMESA (Figure S11B, Supporting Information), but significantly smaller than that of DNA helicase‐MT (Figure S11D, Supporting Information). These observations were consistent with the RMSD values obtained from the MD simulations.

Finally, to understand the energy contribution of each compound, stable binding conformations between 5 and 10 ns of MD simulations were extracted to calculate the MM/GBSA binding free energy (*Δ*G, **Tables** **1** and **2**).^[^ ^26^ ^]^ The 5‐OMESA, MT, and MOS that bound to FtsZ were ranked based on the *Δ*G as follows: FtsZ‐MOS (−35.22 kcal mol⁻^1^), FtsZ‐5‐OMESA (−21.65 kcal mol⁻^1^), and FtsZ‐MT (−21.58 kcal mol⁻^1^) (Table 1). Similarly, MOS exhibited the lowest *Δ*G value when bound to DNA helicase (−23.59 kcal mol⁻^1^), followed by DNA helicase‐5‐OMESA (−14.29 kcal mol⁻^1^) and DNA helicase‐MT (−10.71 kcal mol⁻^1^), indicating the strongest binding affinity of MOS for both targets. These findings are consistent with MOS's in vitro antibacterial activity, further supporting its role in disrupting bacterial function.

**Table 1:** Binding free energies (kcal mol⁻^1^) calculated using the MM‐PBSA method for the *Psa* FtsZ with 5‐OMESA, MT, and MOS during a 10 ns MD simulation.

Compounds	MM/GBSA‐1 (kcal mol⁻^1^)	MM/GBSA‐2 (kcal mol⁻^1^)	MM/GBSA‐3 (kcal mol⁻^1^)	MM/GBSA‐AVE (kcal mol⁻^1^)
5‐OMESA	−16.80 ± 3.51	−18.43 ± 2.05	−29.71 ± 5.35	−21.65 ± 3.64
MT	−23.30 ± 2.58	−20.87 ± 2.76	−20.57 ± 1.68	−21.58 ± 2.34
MOS	−40.24 ± 1.07	−34.95 ± 3.88	−30.46 ± 3.40	−35.22 ± 2.78

*Note*: *n* = 3. Results are shown as the mean ± SD.

**Table 2:** Binding free energies (kcal mol⁻^1^) calculated with the MM‐PBSA method for the *Psa* DNA helicase with 5‐OMESA, MT, and MOS during a 10 ns MD simulation.

Compounds	MM/GBSA‐1 (kcal mol⁻^1^)	MM/GBSA‐2 (kcal mol⁻^1^)	MM/GBSA‐3 (kcal mol⁻^1^)	MM/GBSA‐AVE (kcal mol⁻^1^)
5‐OMESA	−15.65 ± 4.83	−18.45 ± 1.27	−8.77 ± 4.90	−14.29 ± 3.67
MT	−7.94 ± 3.68	−14.10 ± 7.60	−10.09 ± 3.18	−10.71 ± 4.82
MOS	−21.95 ± 4.02	−17.85 ± 2.18	−30.98 ± 3.88	−23.59 ± 3.36

*Note*: *n* = 3. Results are shown as the mean ± SD.

These results collectively support the molecular basis of MOS's antibacterial mechanism. Future studies will further validate these computational predictions through experimental GTP hydrolysis assays and polymerization inhibition studies on FtsZ to confirm MOS's ability to interfere with bacterial cell division.

### 2.6 Synthesis and Characterization of MOS@HPCD

**Figure 7:**
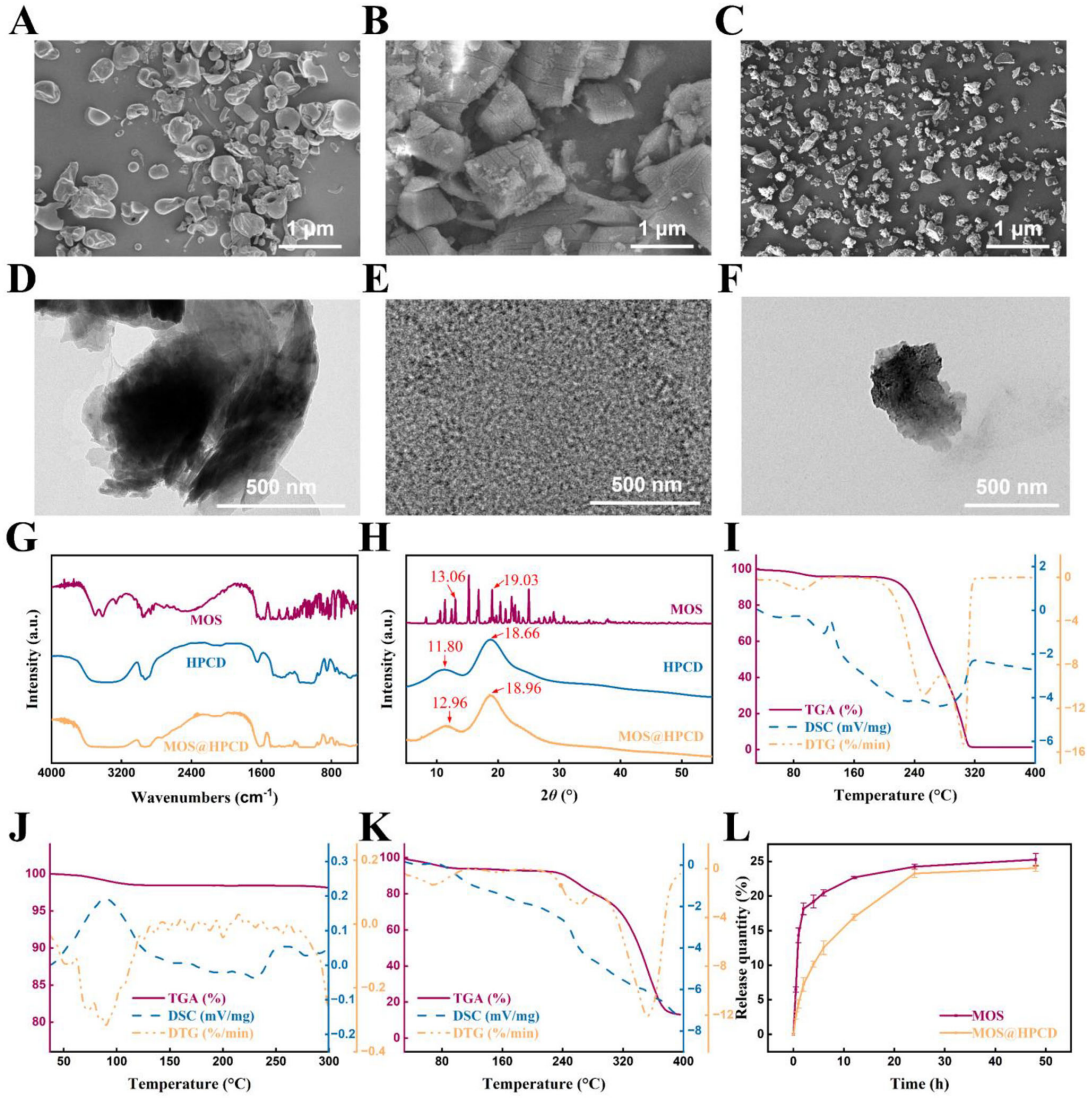
Characterization of HPCD, MOS, and MOS@HPCD. TEM images of A) HPCD, B) MOS, and C) MOS@HPCD. SEM images of D) HPCD, E) MOS, and F) MOS@HPCDG. G) IR spectra of MOS, HPCD and MOS@HPCD; H) XRD patterns of HPCD, MOS and MOS@HPCD; TGA, DSC, and DTG curves of I) MOS, J) HPCD, and K) MOS@HPCD. L) Release profiles of MOS and MOS@HPCD. *n* = 3. Results are shown as the mean ± SD.

Although the ion salt MOS was successfully prepared, its solubility in water was still insufficient for practical application. Generally, nano‐delivery systems are used to improve the solubility of insoluble pesticides. These nanoparticles significantly increase the surface area of the pesticide and improve the contact between the pesticide and solvent, which facilitates the dissolution of insoluble pesticides.^[^ ^27^ ^]^ In this study, the supramolecular nanocarrier, HPCD, with a hydrophobic core and a hydrophilic outer surface, was used to load and generate MOS. The loading efficiency of MOS@HPCD was 65.6 wt.%. This study also revealed that the HPCD loading increased the solubility of MOS in an aqueous solution. As expected, the solubility of MOS@HPCD in water improved (530.0 g L⁻^1^), and it was 8.6‐fold more soluble than MOS (Figure S12, Supporting Information). SEM and TEM analyses demonstrated a smooth surface for HPCD and a dense crystalline sheet‐like structure for MOS (**Figure** **7**A–C). Upon encapsulation by HPCD, MOS formed a block‐like structure with cavities (Figure 7D–F). In the IR spectra (Figure 7G), MOS displayed characteristic peaks of the carboxyl group at 3 261 cm⁻¹, vibrations of C‐H stretching at 2934 cm⁻¹, and a broad peak of the hydroxyl group between 3000 and 3600 cm⁻¹. These were all present in the spectrum of MOS@HPCD. These observations confirm the successful preparation of MOS@HPCD, which proved the suitability of the HPCD cavity to encapsulate hydrophobic molecules. Additionally, the HPCD displayed characteristic peaks at 11.80° and 18.66° in the analysis of XRD pattern.^[^ ^28^ ^]^ Following the loading of MOS in HPCD, a significant shift was observed for these peaks to 12.96° and 18.96°, respectively (Figure 7H). These findings provide compelling evidence for intermolecular interactions between HPCD and MOS. In addition, the HPCD encapsulation significantly reduced the particle size of MOS (Figure S13, Supporting Information). MOS had a particle size of 2 660 nm, while that of MOS@HPCD was 308 nm. This reduction in particle size can be attributed to the ability of HPCD to prevent the MOS crystals from aggregating during the crystallization process.^[^ ^29^ ^]^ In other words, the cavities in the HPCD molecules probably inhibited the formation of larger MOS particles by restricting the growth of MOS crystals, thus, ensuring a finer dispersion of MOS. Encapsulation also provides a protective barrier, which prevents the premature degradation of MOS and improves its thermal stability. Consistent with this hypothesis, the thermogravimetric analysis (TGA) revealed the enhanced heat resistance of MOS@HPCD. The differential scanning calorimetry (DSC) revealed an endothermic peak for MOS at 129.3 °C, which might correspond to its melting point. After 200 °C, the DSC of MOS displayed a concave peak, and a corresponding decrease was found in derivative thermogravimetry (DTG). These findings indicate that the decomposition of MOS commences at this temperature. The DSC analysis of MOS@HPCD showed a smaller endothermic peak near 80 °C, which corresponded to the release of water molecules, and a main endothermic peak at 220 °C, which corresponded to the decomposition of MOS@HPCD. Moreover, in the DTG curve, MOS exhibited a major exothermic peak around 306 °C, while MOS@HPCD displayed a shift in the exothermic peak to 351 °C (Figure 7I–K). This increase in decomposition temperature reflects the role of HPCD in stabilizing MOS by encapsulating it in a more thermally stable matrix. It probably does so by preventing its molecular mobility and exposure to external stresses. In conclusion, the TEM, SEM, XRD, TGA, DSC, DTG, and IR images indicated the successful preparation of MOS@HPCD. The stability of MOS@HPCD was analyzed using a method that involved storage and changes in temperature to determine the system's overall efficacy. The findings indicated that no precipitation, phase separation, or flocculation occurred in MOS@HPCD with different treatments, thus proving the system's robustness (Figure S14 and Table S5, Supporting Information).

### 2.7 Release Kinetics

The study further analyzed the in vitro controlled release performance of MOS and MOS@HPCD until 60 h at a pH of 7.4. As illustrated in Figure 7L, the MOS and MOS@HPCD release rates in the second hour were 18.1% and 7.2%, respectively. Nevertheless, MOS continued to exhibit a rapid release profile and attained a rate of 24.3% at 24 h. Conversely, the rate of MOS@HPCD release decelerated within 24 h. It reached 23.3% after 24 h and remained stable. Although the release quantity of MOS and MOS@HPCD were similar, after the encapsulation of MOS in HPCD, the push‐pull effect of the MOS molecules between HPCD and water enabled the MOS to have a sustained release property. This probably contributed to its prolonged antibacterial activity.

### 2.8 Reduced Contact Angle and Increased Retention of MOS@HPCD in Kiwifruit Leaves

**Figure 8:**
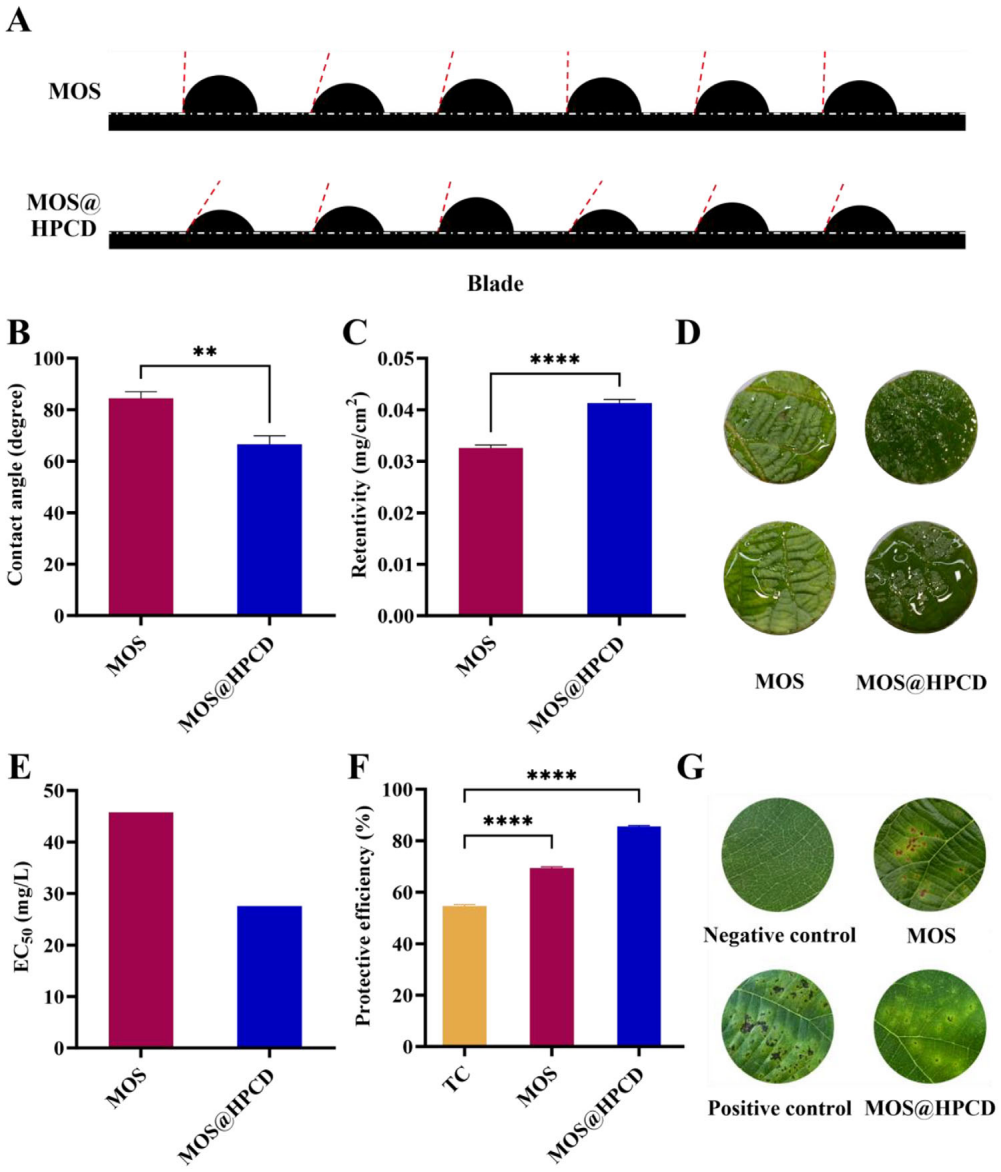
A) Contact angle images of MOS and MOS@HPCD on the blade. The 5 µL of various formulations (final concentration of MOS: 1 mg mL⁻^1^) were dripped onto the blade, and the image of the contact angle was collected (*n* = 6). B) Contact angles of MOS and MOS@HPCD on kiwifruit leaves (*n* = 6). C) Retentivities of MOS and MOS@HPCD on kiwifruit leaves (*n* = 6). D) The practical simulation of MOS and MOS@HPCD on kiwifruit leaves. E) In vitro EC_50_ values of MOS and MOS@HPCD against *Psa* after 48 h. EC_50_ values were determined by probit regression modeling, using inhibition rates from three replicates at 25, 50, 100, 200, and 400 mg L⁻^1^. F) In vivo antibacterial activities of MOS and MOS@HPCD against *Psa* at a concentration of 500 g/ha after 96 h (*n* = 3). G) Protective activities of TC, MOS, and MOS@HPCD on *Psa*‐induced kiwifruit leaf spots. Kiwifruit leaves treated with water alone were served as the negative control; *Psa*‐induced kiwifruit leaf was served as the positive control; MOS and MOS@HPCD were applied at a concentration of 500 g/ha. Data presented as mean ± SEM. Statistical analyses were performed using Student's *t*‐test. **p < 0.01, **** *p* < 0.0001. *Psa*, *Pseudomonas syringae* pv. *actinidiae*.

Typically, efficient deposition and strong adhesion to the leaf surface are crucial for minimizing pesticide loss and improving its utilization efficiency.^[^ ^30^ ^]^ HPCD is an oligosaccharide with a unique cyclic structure, internal hydrophobicity, and external hydrophilicity. Owing to these features, HPCD serves as a carrier to increase the wettability of active ingredients on the leaf surface and reduce the leaf's contact angle.^[^ ^31^ ^]^ Moreover, these features make it easier for pesticide droplets to deposit and adhere to the leaf surface, which reduces the bounce of droplets and improves spray efficiency. These data showed a contact angle of 84.42° for MOS and 66.58° for MOS@HPCD after 10 s of contact with the kiwifruit leaf blade (**Figure** **8**A,B). The decreased contact angle of MOS might be owing to the composition of HPCD, which contains hydrophilic polyhydroxy shells that effectively mitigate the surface tension of droplets. Therefore, these observations suggest that the encapsulation of MOS by HPCD could diminish the surface tension of MOS droplets, thereby facilitating its diffusion and adhesion onto the surface of kiwifruit leaves.

Further supporting these observations, contact angle measurements confirmed that MOS@HPCD significantly reduced surface tension, promoting a more uniform distribution on kiwifruit leaves (Figure 8A–D). This improved wettability is expected to enhance field application efficiency by minimizing pesticide loss due to runoff and improving adhesion to plant surfaces. Additionally, the wettability of plant leaves is a crucial factor that influences the retention rate of pesticides; this feature determines whether the droplets can fully contact and diffuse into the leaves.^[^ ^32^ ^]^ Consequently, it impacts whether the pesticide can exert its desired effect. In this study, after the kiwifruit leaves had been completely soaked in the prepared complex for 30 s, the retentivity of the MOS solution was 32.6 µg cm⁻^2^, whereas that of MOS@HPCD was 41.3 µg cm⁻^2^. This observation indicated a significant increase in MOS@HPCD (1.27‐fold) compared to MOS and demonstrated that kiwifruit leaves are more susceptible to wetting by MOS@HPCD (Figure 8C,D). Therefore, it is anticipated that the application of MOS@HPCD will reduce the surface tension of pesticide droplets and thereby facilitate their spread and adhesion, which results in the efficient control of *Psa*. Furthermore, in vivo assays confirmed that MOS@HPCD‐treated plants exhibited faster healing and stronger disease suppression compared to MOS alone, further validating its potential role in KBC control (Figure 8E–G). The sustained‐release properties of MOS@HPCD contribute to prolonged antimicrobial activity, reducing the need for frequent reapplications and improving long‐term field efficacy. These findings indicate that while MOS itself has strong antibacterial properties, its formulation into MOS@HPCD enhances its practical effectiveness in agricultural applications.

### 2.9 In Vitro and In Vivo Antibacterial Assays against *Psa*

In vitro and in vivo assays were performed to better understand the bactericidal effects of MOS and MOS@HPCD against *Psa* (Figure 8E,F; Figure S15, Supporting Information). The in vitro EC_50_ value of MOS@HPCD against *Psa* was 27.62 mg L⁻^1^ (Figure 8E), which was 1.66‐fold lower than that of MOS (45.83 mg L⁻^1^). MOS encapsulated by HPCD exhibited significantly higher protective efficacy (85.6%) than MOS (69.3%) and TC (54.6%) at 500 g/ha (Figure 8F), demonstrating superior antibacterial efficacy compared to their curative activities (Figure S16, Supporting Information). Quantitative analysis of *Psa* colonization of leaves revealed that intercropping reduced *Psa* abundances in kiwifruit leaves treated with TC, MOS, and MOS@HPCD by 32.1‐55.4% in protective activity assays (Figure S17A, Supporting Information) and by 40.1–57.9% in curative activity assays (Figure S17B, Supporting Information). These findings were further corroborated by in vivo experiments, where MOS@HPCD treatment resulted in faster recovery of the decayed leaves, with much quicker scabbing and healing than in the plants treated with solely MOS after 4 days (Figure 8G). The inhibitory trend is consistent with the in vivo activity. The enhanced in vivo control efficacy of MOS@HPCD against *Psa* is attributed to improved solubility, stronger absorption and penetration, deposit on the leaves and wettability, and sustained release.^[^ ^32^ ^]^

### 2.10 Evaluation of the Safety of MOS and MOS@HPCD

Agrochemicals should ideally exhibit minimal toxicity to non‐target organisms to ensure their suitability for agricultural applications.^[^ ^33^ ^]^ In this study, phytotoxicity in kiwifruit seedlings, as well as the toxicological effects of MOS and MOS@HPCD on zebrafish embryos, human skin fibroblasts (HSF), mice, and rats, were evaluated to comprehensively assess their safety for agricultural application. For phytotoxicity evaluation, kiwifruit seedlings were treated with 500 and 1000 g/ha of MOS and MOS@HPCD, and their growth status was observed over a 21‐day period. No visible symptoms of phytotoxicity, such as leaf yellowing, stunted growth, necrotic, or wilting, were detected in any treated plants (Table S6 and Figure S18, Supporting Information). The leaves remained green and healthy, confirming that MOS and MOS@HPCD are safe for kiwifruit at the tested concentration. Further evaluation of the acute toxicities of MOS and MOS@HPCD on zebrafish embryos at 96 h showed that the lethal concentration at 50% (LC_50_) values of MOS and MOS@HPCD were 588.8 and 1210.8 mg L⁻^1^, respectively (Figure S19, Supporting Information). Among the various compounds tested, MOS and MOS@HPCD had low acute toxicity (10 mg L⁻^1^) on zebrafish embryos, which indicates that they are relatively eco‐friendly. In addition, the 50% inhibitory concentration (IC_50_) value of MOS was 0.27 mg mL⁻^1^ in HSF cells. MOS@HBPCD exhibited even lower cytotoxicity, with an IC_50_ value of 1.58 mg mL⁻^1^ (Figure S20, Supporting Information).

In mice, the lethal dose 50% (LD_50_) value of MOS and MOS@HPCD was 513.9 mg kg⁻^1^ and > 1000 mg kg⁻^1^ (Table S7, Supporting Information), respectively. These values suggest that MOS@HPCD is unlikely to pose safety risks. In addition, MOS and MOS@HPCD in the blood rapidly increased and was then followed by a slower decrease in the Sprague Dawley rats. This study examined the effects of MOS and MOS@HPCD on their metabolism. It found that both MOS and MOS@HPCD are metabolized quicker in female rats than male ones. However, MOS@HPCD (*t*_1/2_ = 0.509 h in male and *t*_1/2_ = 0.454 h in female) was metabolized quicker than the MOS (*t*_1/2_ = 0.927 h in male and *t*_1/2_ = 0.734 h in female) as shown in Figure S21 (Supporting Information). In summary, after the MOS was loaded into HPCD, MOS@HPCD had much lower acute toxicity owing to its core‐shell structure, which reduced damage owing to direct contact in the toxicological models.^[^ ^34^ ^]^

## 3 Conclusion

This study reported the synthesis and application of the organic salt MOS, which has strong antibacterial activity against *Psa*. This study demonstrates that MOS effectively controls KBC through multiple biological mechanisms. ‌Initially, MOS disrupts bacterial cell membranes, increasing permeability and inducing physiological stress. Second, MOS targets key bacterial proteins, FtsZ and DNA helicase, inhibiting their function and leading to bacterial cell death. These mechanisms highlight the potential of MOS as a potent antibacterial agent for KBC management. Furthermore, a nano supramolecular delivery system MOS@HPCD with improved water solubility, absorption, conductivity, transport capacity, and long‐term efficacy was developed for practical application. This system exhibited outstanding in vitro and in vivo antibacterial activities against *Psa*. MOS@HPCD further enhances MOS's solubility, adhesion, and sustained efficacy in planta, improving its real‐world applicability. Thus, MOS and MOS@HPCD, which have no toxic effects on non‐target organisms, such as zebrafish embryos and mice, are promising alternatives to control KBC. By integrating experimental validation with computational modeling, this study provides a comprehensive mechanistic understanding of MOS's antibacterial effects, establishing it as a promising candidate for sustainable plant disease management. However, additional studies should assess the antibacterial activities of other SA salts substituted with MT and the potential of MOS@HPCD in the actual practice and evaluate its long‐term environmental impact under field conditions.

## 4 Experimental Section

### Materials

5‐OMESA (purity, 99%), MT (purity, 99%), and HPCD (purity, 98%) were purchased from Jilin Chinese Academy of Sciences‐Yanshen Technology Co., Ltd (Changchun, Jilin, China). The analytical and high‐performance liquid chromatography solvents used in this study were sourced from Sinopharm (Beijing, China) without purification.

### Preparation of MOS

Approximately 0.01 mol of 5‐OMESA and 0.01 mol of MT were dissolved in 20 mL of ethanol in a 50 mL single‐neck flask, mixed by stirring under 60 °C for 1 h, and then allowed to cool at room temperature. Subsequently, ethanol from the mixture was allowed to evaporate slowly to obtain the colorless MOS crystal (90.2% wt).

### Characterization

The crystal structure data of MT were collected at 170 K on a Rigaku Oxford Diffraction Supernova Dual Source, Copper at Zero equipped with an AtlasS2 CCD (Agilent Technologies, Santa Clara, CA, USA) using Cu Kα radiation (1.54178 Å) via a ω scan mode. The 500 MHz (^1^H NMR) and 125 MHz (^13^C NMR) spectra were collected on a Bruker AVANCE NEO 400 MHz FT‐NMR Spectrometer (Karlsruhe, Baden‐Wuerttemberg, Germany). The elements were analyzed using an elemental UNICUBE analyzer (Langenselbold, Hesse, Germany). The diameter of the crystal was analyzed using a Litesizer™ 500 Particle Analyzer (Graz, Austria), while the IR spectra data were obtained using a JASCO FT/IR‐4600 spectrometer (Gross‐Umstadt, Hessen, Germany). SEM images were obtained using a SU8010 SEM (Hitachi, Tokyo, Japan), while transmission electron microscopic (TEM) images were taken using a FEI Tecnai G2F30 (Hillsboro, TX, USA) operated at 120 kV. TGA, DSC, and DTG were conducted on an STA 449 F3 Jupiter equipment (Netzsch, Selby, Bavaria, Germany). The XRD data were recorded on a Bruker D8 Advance XRD instrument (Karlsruhe, Baden‐Wuerttemberg, Germany).

The solubility of the different compounds was determined utilizing the saturation shake‐flask method.^[^ ^35^ ^]^ First, a concentration‐absorbance calibration curve was constructed for each sample by measuring the absorbance values of serially diluted solutions on a PerkinElmer Lambda 365 spectrophotometer (Waltham, MA, USA). Saturated aqueous solutions of the tested compounds were then prepared by adding excess sample to deionized water, followed by 0.5 h of ultrasonication and 48 h of stirring for equilibration. The saturated aqueous solution was then carefully collected and filtered to create a transparent solution. The solubility of this transparent solution in water was subsequently determined from the calibration curves.

### In Vitro Antibacterial Assay

Nutrient broth (NB) that contained *Psa* was mixed with the NB solvent that contained the tested compounds in a test tube. This inoculant was then incubated for 48 h at 30 ± 1 °C with continuous shaking at 180 rpm. The OD_600_ was measured to assess the growth of the culture.^[^ ^36^ ^]^ Finally, the relative percentage inhibition rates were calculated using the following equation:

(1) $$\mathrm{Inhibition}\mathrm{rates}\left(\%\right)=\frac{\mathrm{CK}-T\;}{\mathrm{CK}}\times \;100$$

where *CK* represents the corrected bacterial growth turbidity value in untreated NB, and *T* represents the corrected bacterial growth turbidity value in treated NB. To better understand the antibactericidal activity of each tested compound, the EC_50_ value was calculated using the log‐probit approach.

### Characterization of the *Psa* Cells Using SEM

Approximately 1.5 mL of *Psa* culture was centrifuged, incubated at the logarithmic phase in pH 7.0 PBS, and then resuspended in 1.5 mL of this buffer. These *Psa* cells were mixed individually with 5‐OMESA, MT, 5‐OMESA+MT (molar ratio of 1:1), or MOS at different concentrations and incubated at room temperature for 4 h. Cells mixed with DMSO were used as the control. After cultivation, the samples were washed three times and then another three times with PBS and immobilized with 2.5% glutaraldehyde at 4 °C for 8 h. The cells were then dehydrated with a graded ethanol series and pure *tert*‐butanol for 10 min each. The samples were finally freeze‐dried, sprayed with gold, and analyzed on a Nova Nano SEM 450 (FEI Ltd., Natural Bridge Station, VA, USA).

### Quantum Mechanical Calculations and Penetration Stimulations

The 3‐D layer‐by‐layer stacking of MOS was described using Diamond software.^[^ ^37^ ^]^ The 2‐D fingerprint plot, the atom‐atom contact contributions, and the 3‐D Hirshfeld dnorm surface of MOS were obtained using the Crystalexplorer program. The IGMH isosurface and ESP of MOS in water were calculated using Multuwf and VMD software.^[^ ^38^ ^]^

The bacterial cell membrane model was constructed with CHARMM‐GUI Membrane Builder tools using a mixture of components found in the supporting materials.^[^ ^39^ ^]^ The initial simulation structures of 5‐OMESA, MT, and MOS were optimized using Discovery studio client software, while the structures and force fields of 5‐OMESA, MT, and MOS were generated via the CHARMM‐GUI Ligand Reader and Modeler component.^[^ ^40^ ^]^

In the initial structure, the 5‐OMESA, MT, and MOS molecules were represented by blue spheres, while the phospholipid molecules were displayed using the stick models. In this study, the different colors represent varying phospholipid molecules. In particular, white indicates phosphatidylglycerol, blue indicates dipalmitoyl phosphatidylcholine, and transparent glass‐like structures indicate the water molecules.^[^ ^41^ ^]^ The topology and force field parameters for the different systems were created using the CHARMM 36 force fields, and the structures of 5‐OMESA, MT, and MOS were generated using the CHARMM general force fields webserver. Approximately 20 units of 5‐OMESA, MT, and MOS systems were placed at ≈1 nm from the membrane surface and represented by blue spheres to indicate their distributions in the simulations, with a box measuring 8 nm × 8 nm × 17 nm.

### Homology Modeling

Two potential *Psa* targets, including FtsZ and DNA helicase, were selected in this study.^[^ ^3^, ^42^ ^]^ The 3‐D structures of FtsZ and DNA helicase were predicted by AlphaFold 3 (https://alphafold.ebi.ac.uk/entry/A0A0Q0FPP7) and https://alphafold.ebi.ac.uk/entry/ F3FBX1, respectively.^[^ ^43^ ^]^ The model confidence of the regions of the predicted binding pockets in the *Psa* FtsZ and DNA helicase was very high, with a per residue model confidence score > 90. Further details regarding the sequences used to create the models are listed in the Supporting Information.

### Molecular Docking

AutoDock Vina 1.2.5 was employed to dock the ligands (5‐OMESA and MT) and ion salt MOS into *Psa* FtsZ and DNA helicase.^[^ ^44^ ^]^ The prepare_receptor4 script of MGLTools 1.4.2 was utilized to prepare the protein and allocate the local charges to Gasteiger, and Open Babel 3.0.0 was used to generate the 3‐D structures of 5‐OMESA, MT, and MOS.^[^ ^45^ ^]^ The docking output conformation was set at 20; the protein binding pocket box size was set at 15 × 15 × 15 and the exhaustivity to 8.

### MD Simulation

The MD simulation was conducted using the Gromacs 2023.2 program.^[^ ^46^ ^]^ The amber99sb‐idln molecular force field was selected for the force field, and the TIP3P water model used the protein and MT, 5‐OMESA, and MOS packed in a cube water box. The truncation of the electrostatic and van der Waals interaction in the simulation was set to 1.0 nm, and the time step was set at 2 fs. Moreover, the PME method was used for the long‐range correction of the electrostatic interaction at 30 °C and 1 bar. Each protein‐ligand complex underwent 10 ns production runs, which were initiated with the same initial structure but with differently randomized initial velocities. Finally, the protein backbone structures from each frame were aligned based on their heavy atom coordinates using MDtraj software to capture the ligand movement and the RMSDs and assess the stability of the protein‐ligand bond.^[^ ^47^ ^]^ For each protein‐ligand complex, the average over the entire simulation (10 ns) was represented as the ligand RMSD.

### Preparation of MOS@HPCD

A concentration of 25 mmol of MOS in 15 mL of ethanol was mixed with 25 mmol of HPCD in 30 mL of ethanol. The mixture was stirred at 35 °C for 4 h at 300 rpm and allowed to evaporate to obtain the HPCD encapsulated MOS (MOS@HPCD, 80.2% yield).

### Encapsulation Efficiency and In Vitro Release of MOS

Dialysis was used to determine the encapsulation efficiency of MOS@HPCD. In this process, the unencapsulated MOS was separated from MOS@HPCD by ultraviolet (UV) spectrophotometry (U‐3010, JEOL Ltd., Tokyo, Japan). Methanol was used to dissolve MOS@HPCD, and its encapsulation efficiency was determined at 277 nm.

Approximately 1 mL of the MOS@HPCD suspension was introduced into a dialysis bag and secured at both ends. The release of MOS in 100 mL of simulated lung fluid [0.203 g MgCl_2_·6H_2_O, 0.298 g KCl, 0.126 g Na_2_HPO_4_, 2.604 g NaHCO_3_, 0.063 g Na_2_SO_4_, 0.368 g CaCl_2_·2H_2_O, 0.574 g CH_3_COONa (sodium acetate), 6.019 g NaCl, and 0.097 g Na_3_H_5_C_6_O_3_·2H_2_O (sodium citrate)] (pH = 7.4) was then studied. The compounds were combined with distilled water and 0.9% sodium chloride in a 1000 mL volumetric flask at 37 °C and stirred at 100 rpm on a desktop thermostat. An identical volume of blank release medium was collected at various time points, and the remaining sample volume was analyzed for the content of pesticide using UV spectrophotometry. The cumulative percentage of MOS release was plotted along the vertical axis and time along the horizontal axis to obtain the release curve.

### Determination of pH Values

The sample was placed into a beaker within the packaging container and diluted 100‐fold with deionized water. The temperature was adjusted to 25 ± 2 °C. The pH meter should be calibrated according to the manufacturer's instructions. Two standard buffer solutions (pH 4.00 and pH 6.86) should be selected for calibration at the specified temperature or using the temperature compensation feature. The temperature of the electrode, rinsing water, and standard buffers was equilibrated to the specified temperature (25 ± 2 °C). After calibrating the instrument, the electrode should be rinsed with distilled water and dried with filter paper. The electrode should be introduced into the sample, and the reading on the pH meter should be stabilized before recording the value. Following the measurement, the electrode should be thoroughly cleaned for future use. The pH value should be reported as the average of three measurements accurate to two decimal places (0.01).

### In Vitro Release of MOS@HPCD

Approximately 1 mL of the MOS@HPCD suspension was introduced into a dialysis bag and secured at both ends. The release of MOS in 100 mL of simulated lung fluid [0.203 g MgCl_2_·6H_2_O, 0.298 g KCl, 0.126 g Na_2_HPO_4_, 2.604 g NaHCO_3_, 0.063 g Na_2_SO_4_, 0.368 g CaCl_2_·2H_2_O, 0.574 g CH_3_COONa (sodium acetate), 6.019 g NaCl, 0.097 g Na_3_H_5_C_6_O_3_ ·2H_2_O (sodium citrate)] was then investigated. These compounds were mixed with distilled water and 0.9% sodium chloride in a 1000 mL volumetric flask at 37 °C and stirred at 100 rpm utilizing a desktop thermostat. An identical volume of blank release medium was removed at various time points, and the remaining sample volume was analyzed using UV spectrophotometry to determine the content of agrochemical. Finally, the cumulative percentage of pesticide release was plotted along the vertical axis and time along the horizontal axis to obtain the pesticide release curve.

### Contact Angles and Retentivity Analysis

The contact angle was measured using an OCA25 Optical Contact Angle Meter (Data Physics Corporation, Riverside, CA, USA) according to the manufacturer's instructions to evaluate the wetting performance of MOS and MOS@HPCD. Approximately 5 µL of each formulation was placed on a leaf blade, and the contact angle image was captured when the droplet was stable. The ellipse fitting algorithm, which assumes that the contour of a water droplet is part of an ellipse, was employed to analyze the contact angle of these compounds. Each measurement was repeated three times. A total of 200 mL of the formulation of MOS@HPCD was taken in a beaker. The leaf blades were weighed on an electronic balance and fully immersed in the prepared solution for 30 s. After their removal from the beaker, the treated blades were weighed until no further droplets were present to drop. The average retentivity was then calculated from the observations of five independent samples using the following equation:

(2) $$\mathrm{Retentivity}\;(\mathrm{mg}/{\mathrm{cm}}^{2})=\frac{A\;-\;B}{S}$$

where *A* represents the blade weight after immersion; *B* represents the blade weight before immersion, and *S* represents the superficial area.

### In Vivo Antibacterial Assay

The protective and curative efficacies of the test compounds (TC, MOS, and MOS@HPCD) in controlling the *Psa*‐induced leaf spot disease were determined by measuring the corrected turbidity (Supplementary Information).^[^ ^3^ ^]^ The plate colony counting method was employed to quantify the *Psa* growth assay (Supplementary Information).^[^ ^48^ ^]^

### Zebrafish Embryos Toxicity

Zebrafish embryos were applied to test the acute toxicities of MOS and MOS@HPCD related to the aquatic organisms (Supporting Information).

### HSF Cytotoxicity

HSF cells (CRL‐2522) were used to evaluate the cytotoxicity of MOS and MOS@HPCD (Supporting Information).

### Single‐Dose Acute Oral Toxicity Analysis

Healthy adult SPF ICR Kunming mice were used to evaluate the oral toxicity of MOS and MOS@HPCD (Supporting Information).

### HPLC‐MS/MS Analysis of MOS and MOS@HPBCD in Sprague Dawley Rats

The MOS and MOS@HPCD in the Sprague Dawley rats were separated and quantified by HPLC‐MS/MS (Supporting Information).

### Statistical Analysis

Statistical analysis of the data was conducted using one‐way analysis of variance (ANOVA) for comparisons among multiple groups and a two‐tailed unpaired Student's *t*‐test for comparisons between two groups, using GraphPad Prism 10.0 (Santiago, CA, USA). *p < 0.05 **p < 0.01, ***p < 0.001, and ****p < 0.0001 progressively higher levels of significance. All data are expressed as means ± standard deviation (SD) from at least three experiments.

## Author Contributions

X.D. and Q.B. contributed equally to this work. X.D. and L.B. conceptualized and conducted this research. X.D., T.L., and M.Z. synthesized and characterized MOS and MOS@HPCD. Q.B. and Y.Z. performed biological activity, homology modeling, and MD simulation experiments. X.D. and J.Z. contributed materials. X.D. and J.Z. wrote the manuscript. L.Z., J.Z., and L.B. supervised the project. All the authors provided input and reviewed the manuscript. M.Z. evaluated the long‐term stability and cytotoxicity test. L.X. completed the acute toxicity and pharmacokinetic tests. All the authors provided input to the manuscript and reviewed it.

## Conflict of Interest

The authors declare no conflict of interest.

## Supporting information



Supporting Information

Supporting Information

Supporting Information Video 1

Supporting Information Video 2

Supporting Information Video 3

## Data Availability

CCDC 2370697 contains the supplementary crystallographic data for this paper. These data can be obtained free of charge from The Cambridge Crystallographic Data Centre via www.ccdc.cam.ac.uk/data_request/cif. The data on membrane penetration and docking are available at https://github.com/xyj13771316308/MOS.git.
