# Supplementary material for: A Supramolecular Material for Controlling Kiwifruit Bacterial Canker
Source: Adv Sci (Weinh). 2025 May 24;12(31):e14752. doi: 10.1002/advs.202414752 (PMC12376517; doi:10.1002/advs.202414752)
Supplement: Supplementary file 1 — Supporting Information [file ADVS-12-e14752-s005.docx]

**A Supramolecular Material for Controlling Kiwifruit Bacterial Canker**

*Xile Deng, Qiang Bian,* *Mingqing Zhou, Le Xie, Jichuang Zhang, Tianqi Liu, Yizhuo Zhang, Li Zhang^*^, Jiaheng Zhang^*^, and Lianyang Bai^*^*

X. L. Deng, L. Y. Bai

Hunan Academy of Agricultural Sciences, Changsha 410125, China

E-mail: lybai@hunaas.cn

J. C. Zhang, T. Q. Liu, J. H. Zhang

School of Material Sciences and Engineering, Harbin Institute of Technology (Shenzhen), Shenzhen 518055, China

E-mail: zhangjiaheng@hit.edu.cn

Q. Bian

National Pesticide Engineering Research Center (Tianjin), College of Chemistry, Nankai University, Tianjin 300071, China

Y. Z. Zhang

Department of Microbiology, College of Life Sciences, Nankai University, Tianjin 300071, China

L. Zhang

Department of Applied Chemistry, College of Science, China Agricultural University, Beijing, 100193, China

Email: [zhang_li@cau.edu.cn](mailto:zhang_li@cau.edu.cn)

M. Zhou

Department of Chemistry, College of Sciences, Northeastern University, Shenyang, 110819, China

L. Xie

Department of Neurology, Hunan Academy of Chinese Medicine Affliated Hospital (Hunan Hospital of Integrated Traditional Chinese and Western Medicine), Changsha, 410006, China

**Pages**

[*Psa* FtsZ protein and DNA helicase sequences 4](#_Toc197255699)

[In Vivo Antibacterial Assay 5](#_Toc197255700)

[Security Evaluation 6](#_Toc197255701)

[Supplementary 2-D fingerprint plot and atom–atom contact contributions of 5-OMESA 11](#_Toc197255702)·

[Supplementary 2-D fingerprint plot and atom–atom contact contributions of MT 12](#_Toc197255703)

[Supplementary orderliness 13](#_Toc197255704)

[Supplementary lipid areas 14](#_Toc197255705)

[Supplementary thickness 15](#_Toc197255706)

[Supplementary minimum distances 16](#_Toc197255707)

[Supplementary numbers of hydrogen bonds 17](#_Toc197255708)

[Supplementary RMSD 18](#_Toc197255709)

[Supplementary RMSF 19](#_Toc197255710)

[Supplementary Rg 20](#_Toc197255711)

[Supplementary free energy landscapes 21](#_Toc197255712)

[Supplementary water solubilities 23](#_Toc197255713)

[Supplementary particle size 24](#_Toc197255714)

[Supplementary physical appearance 25](#_Toc197255715)

[Supplementary in vitro antibacterial activity assay 26](#_Toc197255716)

[Supplementary curative activity 27](#_Toc197255717)

[Supplementary *Psa* abundances 28](#_Toc197255718)

[Supplementary kiwifruit security images 29](#_Toc197255719)

[Supplementary acute toxicity on zebrafish embryos at 96 h 30](#_Toc197255720)

[Supplementary cytotoxicity evaluation 31](#_Toc197255721)

[Supplementary pharmacokinetic experiments in rats 32](#_Toc197255722)

[Supplementary crystal data and structure refinement 33](#_Toc197255723)

[Supplementary elemental analysis 35](#_Toc197255724)

[Supplementary hydrogen bonds of MOS 36](#_Toc197255725)

[Supplementary docker energies 37](#_Toc197255726)

[Supplementary stability analysis 38](#_Toc197255727)

[Supplementary evaluation of safety in kiwifruit 39](#_Toc197255728)

[Supplementary acute toxicity toward mice 40](#_Toc197255729)

[Supplementary transmembrane process of 5-OMESA 41](#_Toc197255730)

[Supplementary transmembrane process of MT 42](#_Toc197255732)

[Supplementary transmembrane process of MOS 43](#_Toc197255733)

[References 44](#_Toc197255735)

*Psa* FtsZ protein and DNA helicase sequences

*Psa* FtsZ Protein sequence:

MFELVDNVPQSPVIKVIGVGGGGGNAVNHMVKSNIEGVEFICANTDAQALKNIGARTILQLGTGVTKGLGAGANPEVGRQAALEDRERIAEVLQGTNMVFITTGMGGGTGTGAAPIIAEVAKEMGILTVAVVTRPFPFEGRKRMQIADEGIRMLSESVDSLITIPNEKLLTILGKDASLLSAFAKADDVLAGAVRGISDIIKRPGMINVDFADVRTVMSEMGMAMMGTGCASGPNRAREATEAAIRNPLLEDVNLQGARGILVNITAGPDLSLGEYSDVGSIIEAFASEHAMVKVGTVIDPDMRDELHVTVVATGLGAKIEKPVKVIDNTLQTTQQAPAQQASRQEAPSVNYRDLDRPTVMRNQAHAGATAAAKMNPNDDLDYLDIPAFLRRQAD.

*Psa* DNA helicase sequences:

MSRELYSLEAEHGLLGALLLDASLFDAITARITTADFAYDDNAAMYQAIIDTHAAGQPVDVVTVGFAYPDLPSGERTLAYASEIAKNIPSTANWAGYQRIVLERSALRRVVEAAEVIKDSASESLPVADIIALAQQATADLRDLGAPDRKDYYKYSEVLPGVVDGIDSRFNGAAQLGHETGLKDLDELIRGLRKKNMIVIAGLPGSGKTSLGVQIAQKIACTDNGVGLIVSMEMTKEELVTRGLASVGGISLTRIDQGHTLQDDDWPRLTSAVNVLQNSKLFVCDEEGMTAARIRSTARQVQRKEGLSIVVVDYIGLIAAEGAGQNRTLELGKISTSLKNMAKELDVPVIVLAQLNRGSTNRTDKKPRPSDLRDSGQIEADADVVILVHRDPDSEEGQNGVT

In Vivo Antibacterial Assay

*Protective and Curative Activity Assay*: The antibacterial efficacy of MOS and MOS@HPCD against *Psa* was evaluated using a detached leaf disc assay.^[1]^ Healthy kiwifruit leaves were collected and surface-sterilized in 0.6% sodium hypochlorite (NaOCl) for 5 min, followed by three rinses with sterile distilled water. Leaf discs (1.2 cm in diameter) were punched and used for both protective and curative assays. For protective activity assay, leaf discs were pretreated with distilled water (positive control) or 500 g/ha of the test compounds (thiodiazole copper (TC), MOS, and MOS@HPCD) and incubated at 95% relative humidity in an artificial climate chamber (16 h light at 10 °C and 8 h dark at 16 °C) for 24 h. Subsequently, 10^4^ colony-forming units (CFU)/mL of *Psa* was infiltrated into the leaf discs under vacuum (0.1 MPa for 5 min). Kiwifruit leaf only with water was served as the negative control. For curative activity assay, leaf discs were first infiltrated with 10⁴ CFU/mL of *Psa* under vacuum (0.1 MPa for 5 min). After 24 h, the infected discs were sprayed with distilled water or 500 g/ha of the test compounds (TC, MOS, and MOS@HPCD). For both assays, leaf discs were incubated under the same controlled conditions. Four days post-treatment, lesion areas were measured using ImageJ software, and antibacterial efficacy was calculated using the following formula:

Control effect (%) = $\frac{CK-T}{CK}\text{× 100 }\text{\%}$ (**1**)

where *CK* represents the mean lesion area in untreated samples, and *T* represents the mean lesion area in treated samples. Experiments were performed in triplicate.

*Colony Counting Method for Psa Detection*: To quantify bacterial colonization, each infected leaf disc from the curative and protective assays were rinsed thoroughly in sterile water and homogenized in 200 µL sterile water using sterile metal beads and a tissue lyser. To quantify viable bacteria, serial 10-fold dilutions of leaf homogenates were plated onto LB agar plates supplemented with appropriate antibiotics, incubated at 30 °C for 24 h, and CFU were counted. Bacterial load was calculated as CFU per cm² leaf tissue and expressed as log_10_ CFU/cm².

Security Evaluation

*Evaluation of Phytotoxicity in Kiwifruit*: To assess the phytotoxicity of MOS and MOS@HPCD, a total of three leaves from each kiwifruit seedling that were growing in unison were selected for the treatment and there were four replicates for each treatment. The seedlings were grown in a controlled greenhouse environment at 22-26 °C, with 75-85% relative humidity and a 16 h light/8 h dark photoperiod. Each treatment consisted of foliar spraying with 500 and 1000 g/ha of MOS and MOS@HPCD, with distilled water serving as the control. After 21 days, the treated plants were carefully examined for signs of phytotoxicity, including leaf chlorosis, necrosis, wilting, growth retardation, curling, or morphological deformities. Phytotoxicity symptoms were evaluated using a standard grading system, where "-" indicates no toxicity, "+" represents mild and recoverable toxicity, and "++" or higher indicates significant toxicity with potential growth inhibition. The number of leaves was then calculated. Digital images of treated seedlings were taken to document any visible changes.

*Security Evaluation toward* zebrafish embryos: The acute toxicities of MOS and MOS@HPCD on zebrafish embryos at 96 h post-fertilization (hpf) were examined. At 2 hpf, the zebrafish embryos were randomly placed in 2 mL test solutions at varying concentrations in a 24-well plate. These embryos were incubated with the tested compounds for 96 h to determine their LC_50_. The concentrations of MOS were set at 200, 525, 550, 575, 600, and 650 mg/L, while those of MOS@HPCD were set at 1,150, 1,175, 1,200, 1,225, 1,250, and 1,275 mg/L. All the test solutions were prepared in water, and a mixture of 0.75 mmol/L Na^+^, 2 mmol/L Ca^2+^, 0.5 mmol/L Mg^2+^, 0.074 mmol/L K^+^, and 0.01% acetone was used as the control. The plates were covered with transparent lids to prevent evaporation during the experiment and transferred to an incubator at 27 ± 1 °C with a 14:10 h light/dark photoperiod. The number of dead embryos and the state of embryonic development were examined daily. The experiments were conducted in triplicate using 10 embryos per replicate of each concentration.

*Security Evaluation toward* *Human Skin Fibroblasts (HSF)*: Human skin fibroblast cells (CRL-2522) were purchased from Guangdong Boxi Biotechnology Co., Ltd (Dongguan, Guangdong, China). The cells were seeded in 96-well plates and cultured in Minimum Essential Medium Eagle (MEM, Hyclone, Logan, UT) with Earle's balanced salt and L-glutamine, which was supplemented with 10% fetal bovine serum (FBS, Invitrogen, Carlsbad, CA, USA) and 1% penicillin/streptomycin (PS, Invitrogen). The cells were cultured at 37ºC in a 5% CO_2_ incubator. After 24 h, the cells were washed twice with 100 μL of serum-free MEM (1% PS) and incubated with 100 μL of different concentrations of pGO-5 and GS suspensions in serum-free MEM (1% PS). The pGO-5 and GS particles used for the viability assays were washed five times with serum-free MEM (1% PS). After a 24 h period of exposure, the cells were washed twice with 100 μL of serum-free MEM and then incubated with 100 μL of 0.5 mg/mL methyl thiazolyl diphenyl-tetrazolium bromide (MTT, Invitrogen). The cells were then incubated with this solution for 2 h at 37 °C in a 5% CO_2_ incubator. The media that contained the MTT was then removed, and the insoluble purple formazan crystals produced by the live cells were dissolved in 100 μL of dimethyl sulfoxide (DMSO, Sigma-Aldrich, St. Louis, MO). The plate was then placed on a rocking shaker for a minimum of 20 min. A volume of 80 μL of the purple DMSO solution was transferred to a new 96-well plate for each well. This was necessary because residual pGO-5 or GS can affect the absorbance values at 490 nm. The absorbance of the stain produced was then monitored at 490 nm using an iMark microplate reader (Bio-Rad, Hercules, CA). The cell viability was determined by its mitochondrial activity, which was calculated using equation **(1)**. Cells not exposed to particles were used as a control, and cell-free control experiments were performed to ascertain whether the GO and GS reacted directly with the MTT reagents. Typically, pGO-5 and GS particles with different concentrations (3.125, 200 μg/mL) were suspended in 1 mL of 0.5 mg/mL of MTT (in MEM). Following incubation at 37 °C in 5% CO_2_ for 2 h, the pGO-5 and GS particles were washed with PBS. A volume of 1 mL of DMSO was added to redisperse the pelleted particles to determine if any insoluble formazan had formed during the incubation. The suspended pGO-5 and GS particles were then centrifuged again, and the DMSO extract was measured at 490 nm. This was used to determine whether the MT Treagen interacted with the pGO-5 and GS particles.

*Relative cell viability* (%) = $\frac{\text{TestOD}\text{490 nm}}{\text{Neg}\text{OD}\text{490 nm}}\text{ × }\text{100 \%}$ **(1)**

where *TestOD_490 nm_* represents the 490 nm values for the CRL-2522 cells, which were treated with MOS and MOS@HPCD, and *NegOD_490 nm_* represents the 490 nm values of the untreated cells. The IC_50_ (half maximal inhibitory concentration) values were calculated using the log-probit approach. There were at least three replicates for all the experiments.

*Single-dose acute oral toxicity analysis of MOS and MOS@HPCD*: There were 20 healthy adult SPF ICR mice in each treatment, with an average weight of 20 ± 2 g. The sample size was divided equally between 10 males and 10 females in a treatment, and the animals were provided by the Hunan SJA Laboratory Animal Co., Ltd (430727251100062276) (Changsha, Hunan, China). Ethical approval for this study was obtained from the Experimental Animal Ethics Committee of the Hunan Academy of Chinese Medicine (SY2025-0004; Changsha, Hunan, China). The laboratory animal production license number, quality certificate number, and use license number were SYXK (Xiang) 2024-0015. The acute oral toxicity experiment was conducted by the Hunan Academy of Chinese Medicine in accordance with the provisions of the national food safety standard (GB 15193.3-2014). The ambient temperature and relative humidity were maintained at 22-25 °C and 57%-70%, respectively. The animals were deprived of food for 6 h prior to the experiment. However, there were no restrictions on their consumption of water. The cell activity of the GXDK6 sample solution was observed by staining with 0.1% Loeffler's methylene blue stain, and the experimental process from sample solution preparation to animal gavage was completed within 6 h. The GXDK6 sample solution (4.0 × 10^9^ CFU/mL, 1,200 mg/mL) was administered at a rate of 20 mL/kg of mouse body weight (BW). The dose administered was 400 mg/kg BW. The BW and mortality were observed during the 14-d experimental period. The animals were euthanized using isoflurane and then dissected for observation. The animals were provided with ad libitum access to food and water. The food, water, and feed were consistent with the national standards and regulations outlined in GB 14925-2010, GB 5749-2006, GB 14924.1-2001, GB 14924.2-2001, and GB 14924.3-2010. There were at least three replicates for each experiment.

*LC-MS/MS analysis of MOS and MOS@HPCD*: Sprague Dawley rats (average body mass 180 ± 20 g) were procured from the Hunan SJA Laboratory Animal Co., Ltd (430727251100062358). Ethical approval for this study was obtained from the Experimental Animal Ethics Committee of the Hunan Academy of Chinese Medicine (SY2025-0003). The animals were kept in a controlled environment with unrestricted access to food and water and a 24 h light-dark cycle. All the animal research was conducted using approved and monitored guidelines. The animals were euthanized with isoflurane after the study had been completed. This chemical causes little distress. The blood samples collected were used for subsequent research.

A high-pressure liquid chromatography tandem mass spec (HPLC-MS/MS) system composed of an HPLC (Agilent 1290, Agilent Technologies, Santa Clara, CA, USA) and a triple-quadrupole mass spectrometer (AB SCIEX QTRAP 4500) operating in the electrospray ionization (ESI) mode was used to analyze MOS. A ZORBAX RRHD Eclipse plus C18 column (3.0×100 mm id, 1.8 μm particle size) was utilized < 30 °C. The flow rate was set at 0.2 min/L with an injection volume of 2.0 μL. The mobile phase consisted of (A) acetonitrile and (B) ultrapure water. The elution steps were as follows: 90% of solvent B from 0 to 1.0 min; 90-10% from 1.0 to 3.0 min; 10% from 3.0 to 5.0 min; 10 to 90% from 5.0 to 6.0 min; and 90% from 6.0 to 8.0 min.

MS was performed in the positive Electro Spray Ionization (ESI) mode with multiple reaction monitoring (MRM). Nitrogen was used as the curtain, nebulizer, and collision gas (pressure 35, 45, and 45 psi, respectively). The source parameters for the ion spray voltage and source temperature were 4.5 kV and 550 ºC, respectively. The half-life ($\text{t}_{\text{1/2}}$) was calculated using the following equation:

$\text{t}_{\text{1/2}}\text{ }\text{= lg2 ×}\text{ }\frac{\text{2.303}}{\text{K}_{\text{e}}}\text{=}\text{ }\text{0.301 ×}\text{ }\frac{\text{2.303}}{\text{K}_{\text{e}}}\text{ }\text{=}\text{ }\frac{\text{0.693}}{\text{K}_{\text{e}}}$ **(2)**

where K_e_ is the excretion rate constant.

Supplementary 2-D fingerprint plot and atom–atom contact contributions of 5-OMESA


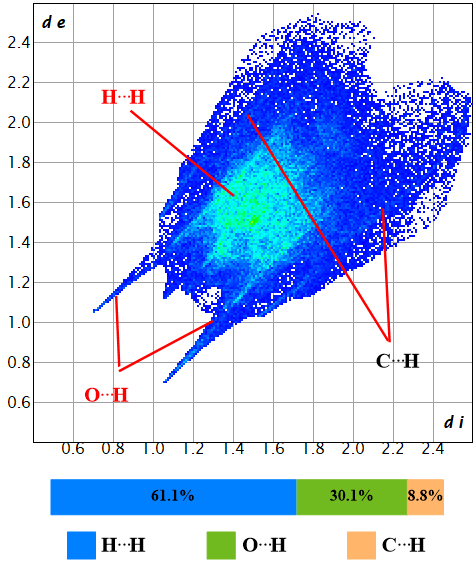


**Figure S1.** 2-D fingerprint plot and atom–atom contact contributions of 5-OMESA.

Supplementary 2-D fingerprint plot and atom–atom contact contributions of MT


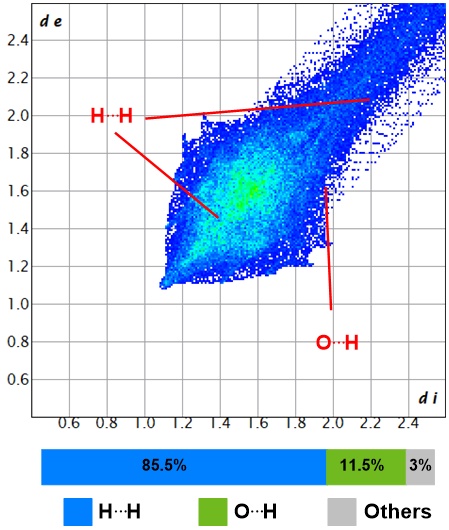


**Figure S2.** 2-D fingerprint plot and atom–atom contact contributions of MT.

Supplementary orderliness


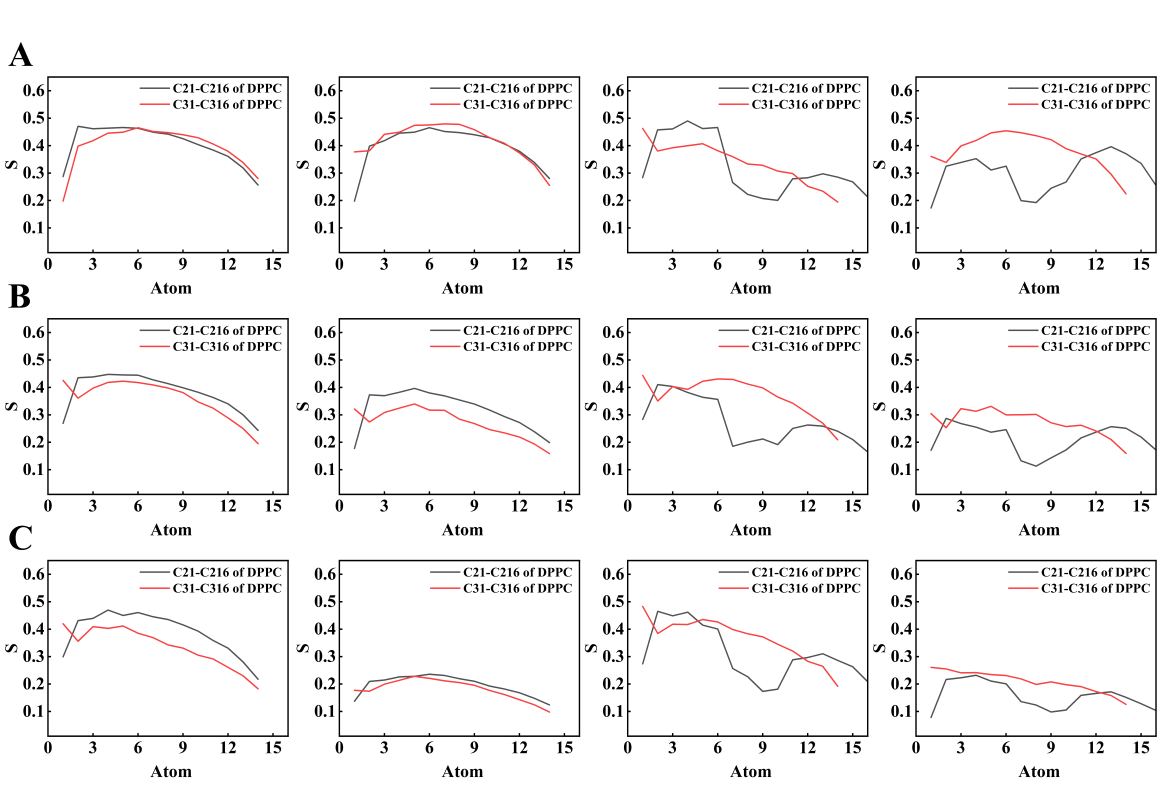


**Figure S3.** Phospholipid tail order parameter analysis of phospholipid molecular tails in three systems: A) 5-OMESA, B) MT, and C) MOS. The phospholipid tail order parameter was assessed by the S_z parameter, where values close to 1 indicate a highly ordered state, while values close to 0 indicate a disordered state. DPPC: 1,2-dihexadecanoyl-rac-glycero-3-phosphocholine. C21-C216 of DPPC: the carbon atoms 1-16 on the C2 chain of DPPC; C31-C316 of DPPC: the first to 16^th^ carbon atoms on the C3 chain of DPPC.

Supplementary lipid areas


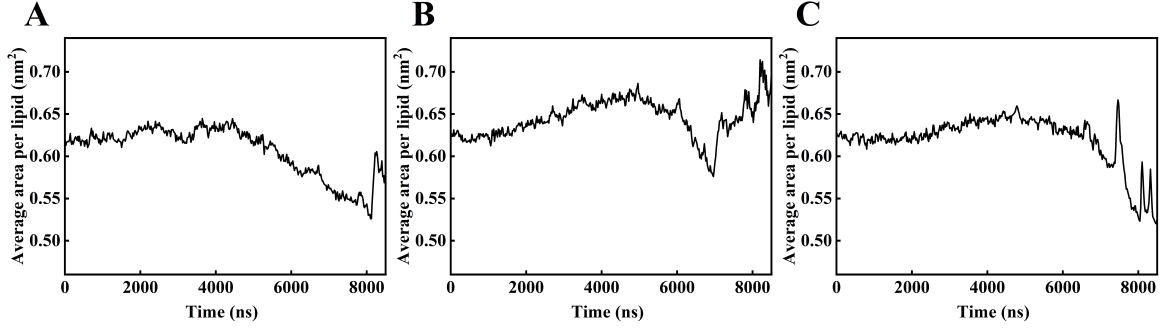


**Figure S4.** Changes in the phospholipid area per lipid of the *Psa* cell membranes have been observed to stimulate osmotic processes in the membrane lipid chaperones. A) 5-OMESA, B) MT, and C) MOS of the *Psa* cell membrane.

Supplementary thickness


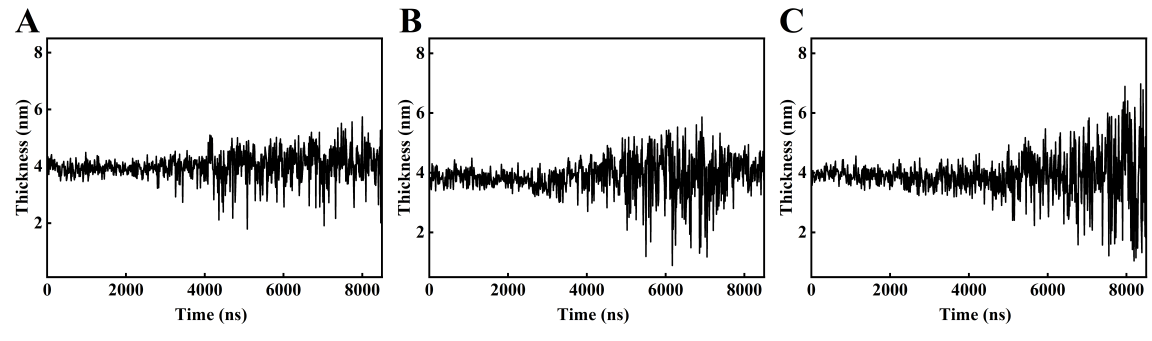


**Figure S5.** Changes in the cell membrane thickness with time in the *Psa* cell membrane lipid chaperones with A) 5-OMESA, B) MT, and C) MOS.

Supplementary minimum distances


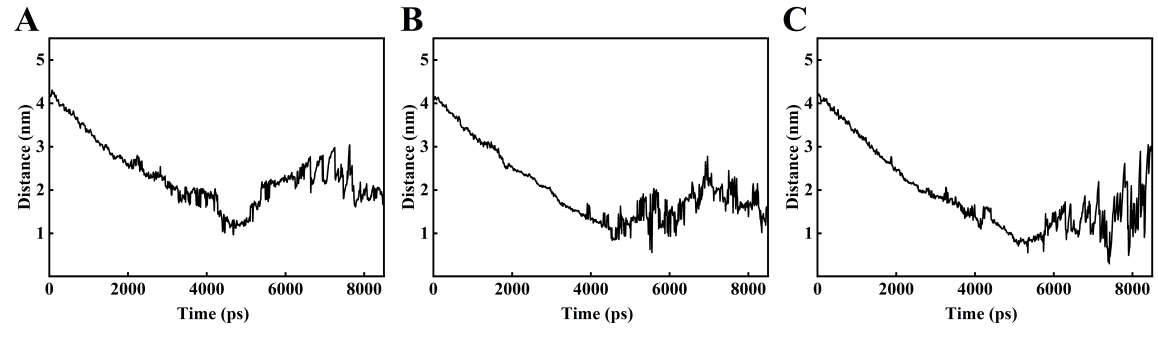


**Figure S6.** The changes in center-of-mass distance with time during the penetration of *Psa* cell membrane by A) 5-OMESA, B) MT, and C) MOS.

Supplementary numbers of hydrogen bonds


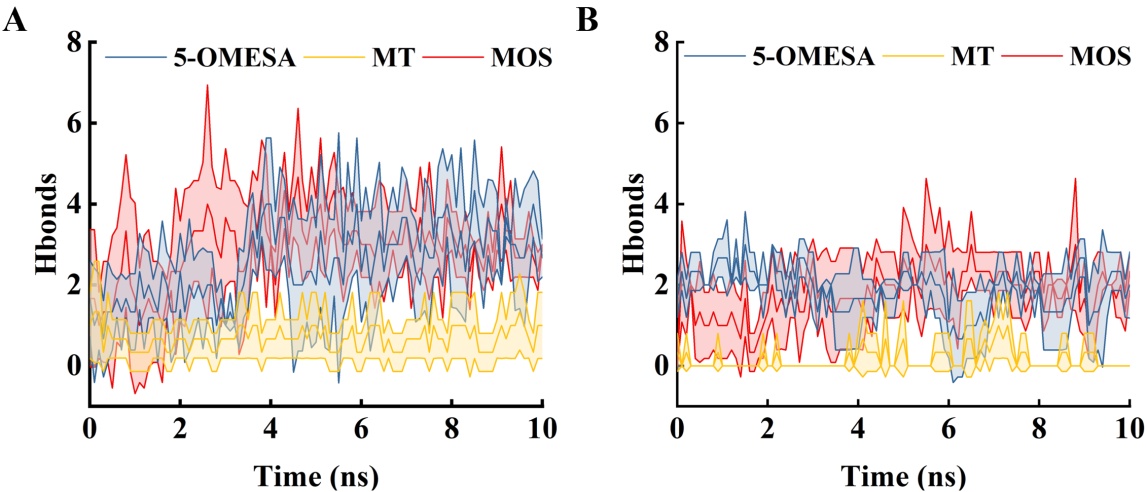


**Figure S7.** Numbers of hydrogen bonds between the protein-ligand complexes throughout the simulations. A: *Psa* FtsZ with 5-OMESA, MT, and MOS. B: *Psa* DNA helicase with 5-OMESA, MT, and MOS. Red: MOS; blue: 5-OMESA; yellow: MT.

Supplementary RMSD


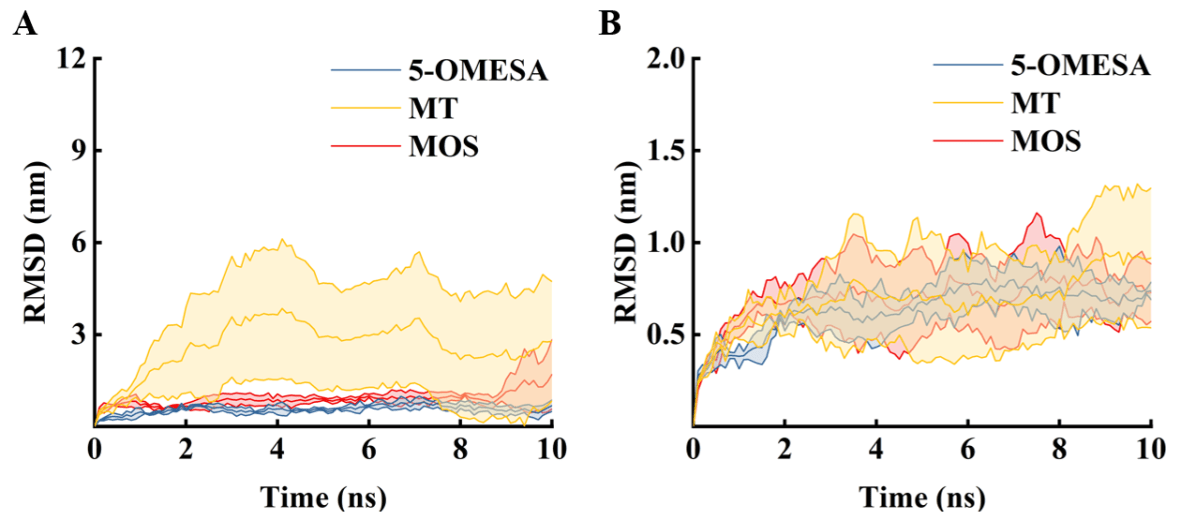


**Figure S8.** Root mean square deviation (RMSD) values for the receptors A) FtsZ and B) DNA helicase systems in *Psa* bound with the ligands 5-OMESA, MT, and MOS, respectively, with respect to 10 ns molecular dynamics. Red: MOS; blue: 5-OMESA; yellow: MT.

Supplementary RMSF


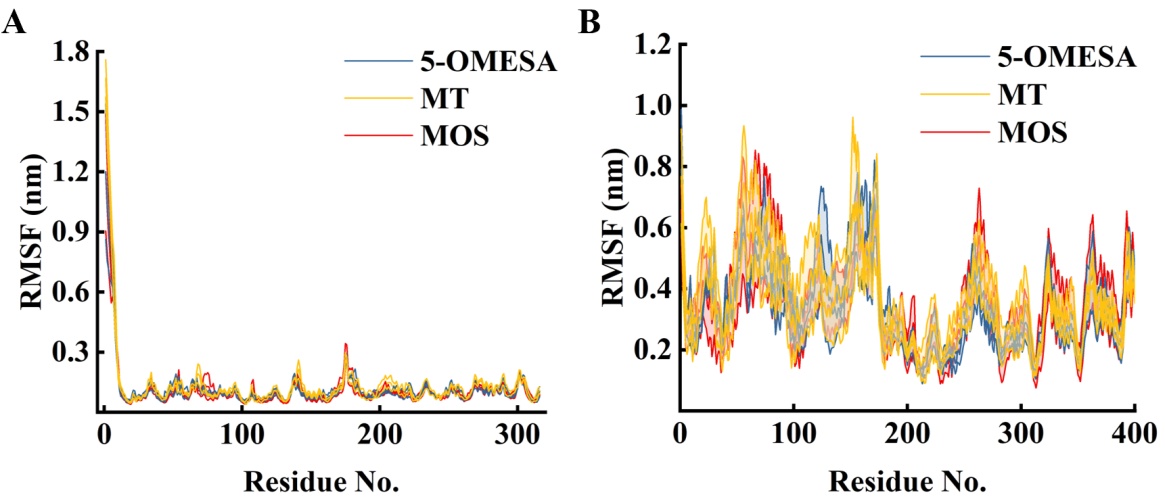


**Figure S9.** Root mean square fluctuation (RMSF) plots of A) FtsZ and B) DNA helicase in *Psa*, bound with 5-OMESA, MT, and MOS during the simulations at 10 ns. Red: MOS; blue: 5-OMESA; yellow: MT.

Supplementary Rg


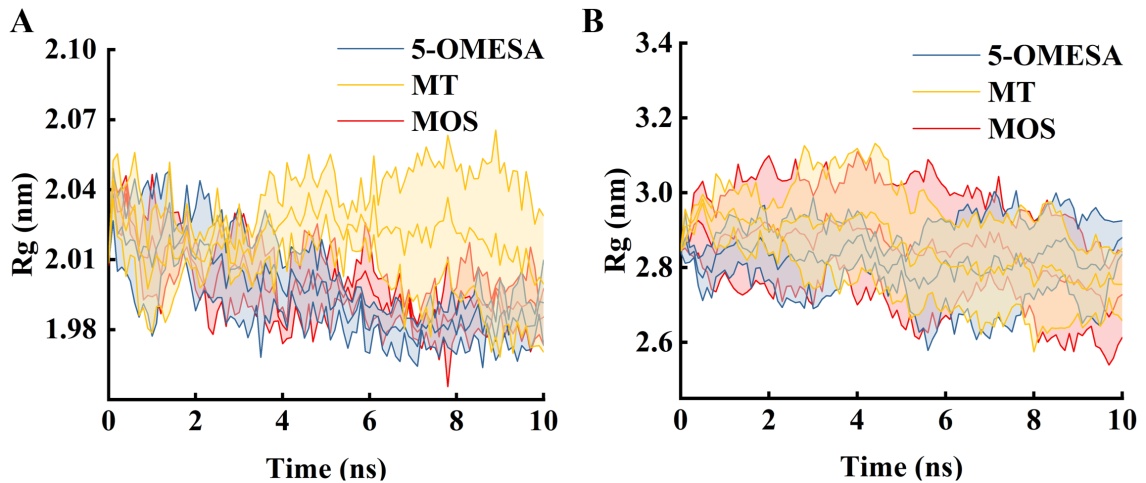


**Figure S10.** Radius of gyration (Rg) of A) FtsZ and B) DNA helicase in *Psa* bound with 5-OMESA, MT, and MOS during the simulations at 10 ns. Red: MOS; blue: 5-OMESA; yellow: MT.

Supplementary free energy landscapes

| **A** |  |  |
| --- | --- | --- |
| **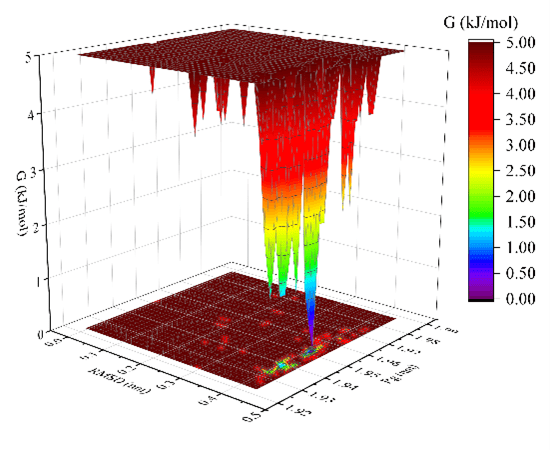** | 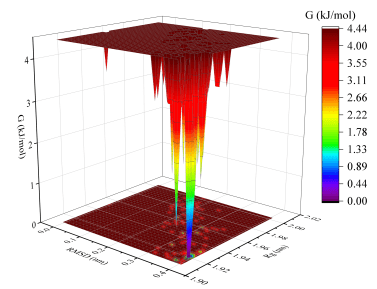 | **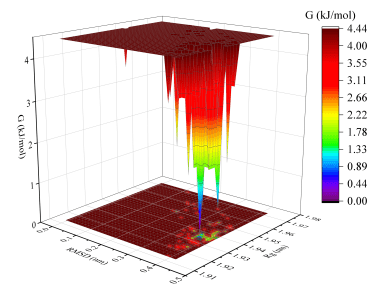** |
| **B** |  |  |
| 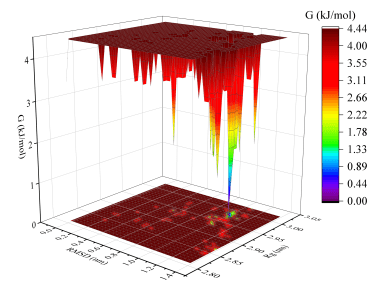 | **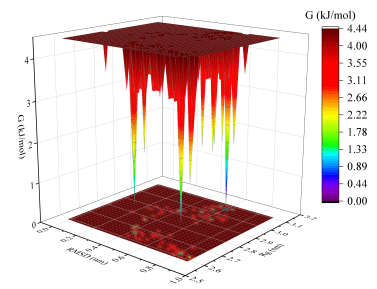** | **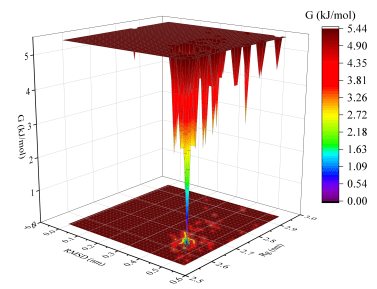** |
| **C** |  |  |
| **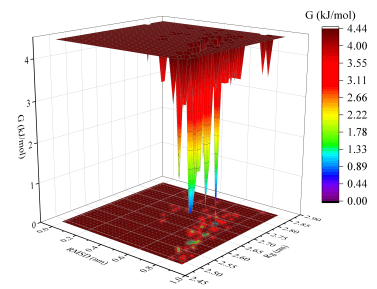** | 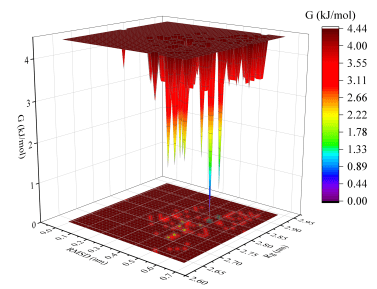 | 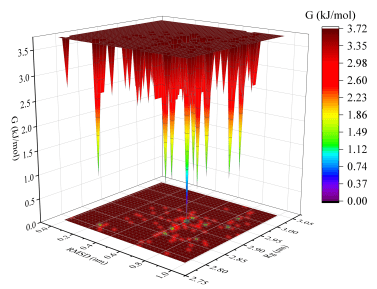 |
| **D** |  |  |
| 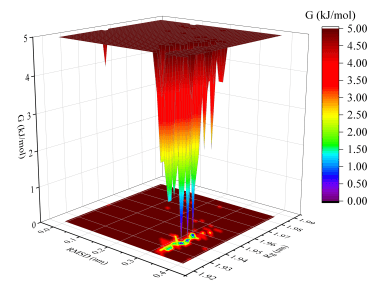 | **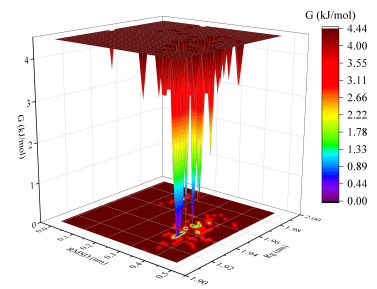** | **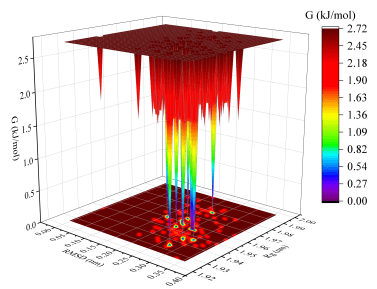** |
| **E** |  |  |
| 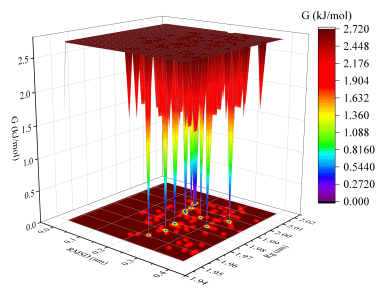 | **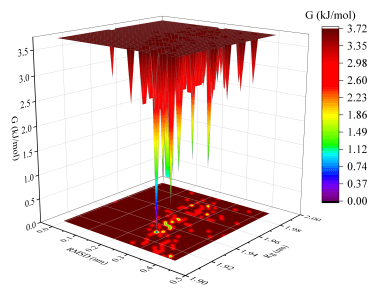** | **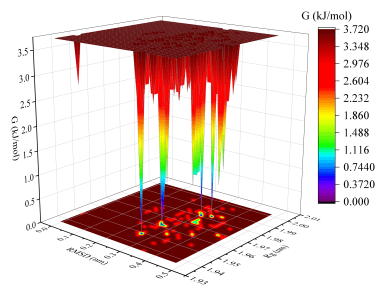** |
| **F** |  |  |
| **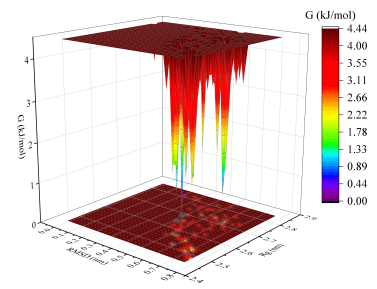** | **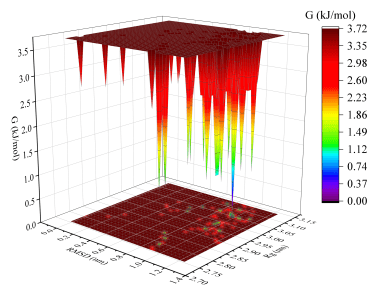** | **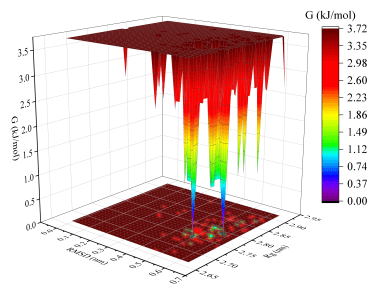** |

**Figure S11.** Three replicates of the free energy landscapes of FtsZ and DNA helicase in *Psa* bound with 5-OMESA (A: FtsZ, B: DNA helicase), MT (C: FtsZ, D: DNA helicase), and MOS (E: FtsZ, F: DNA helicase) during the simulations at 10 ns. *n* = 3.

Supplementary water solubilities





**Figure S12.** Water solubilities of MOS, and MOS@HPCD. *n* = 3. Data are the mean ± SD. Red: MOS, yellow: MOS@HPCD.

Supplementary particle size





**Figure S13.** Particle sizes of MOS and MOS@HPCD. *n* = 3. Data are the mean ± SD. Red: MOS, yellow: MOS@HPCD.

Supplementary physical appearance

**
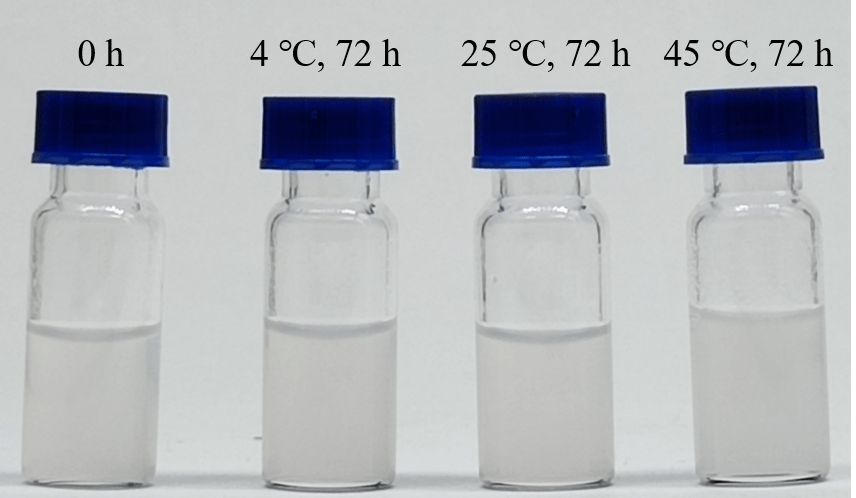
**

**Figure S14.** The physical picture of MOS@HPCD after temperature changes (4 °C, 25 °C, and 45 °C) and storage for 72 h.

Supplementary in vitro antibacterial activity assay

**
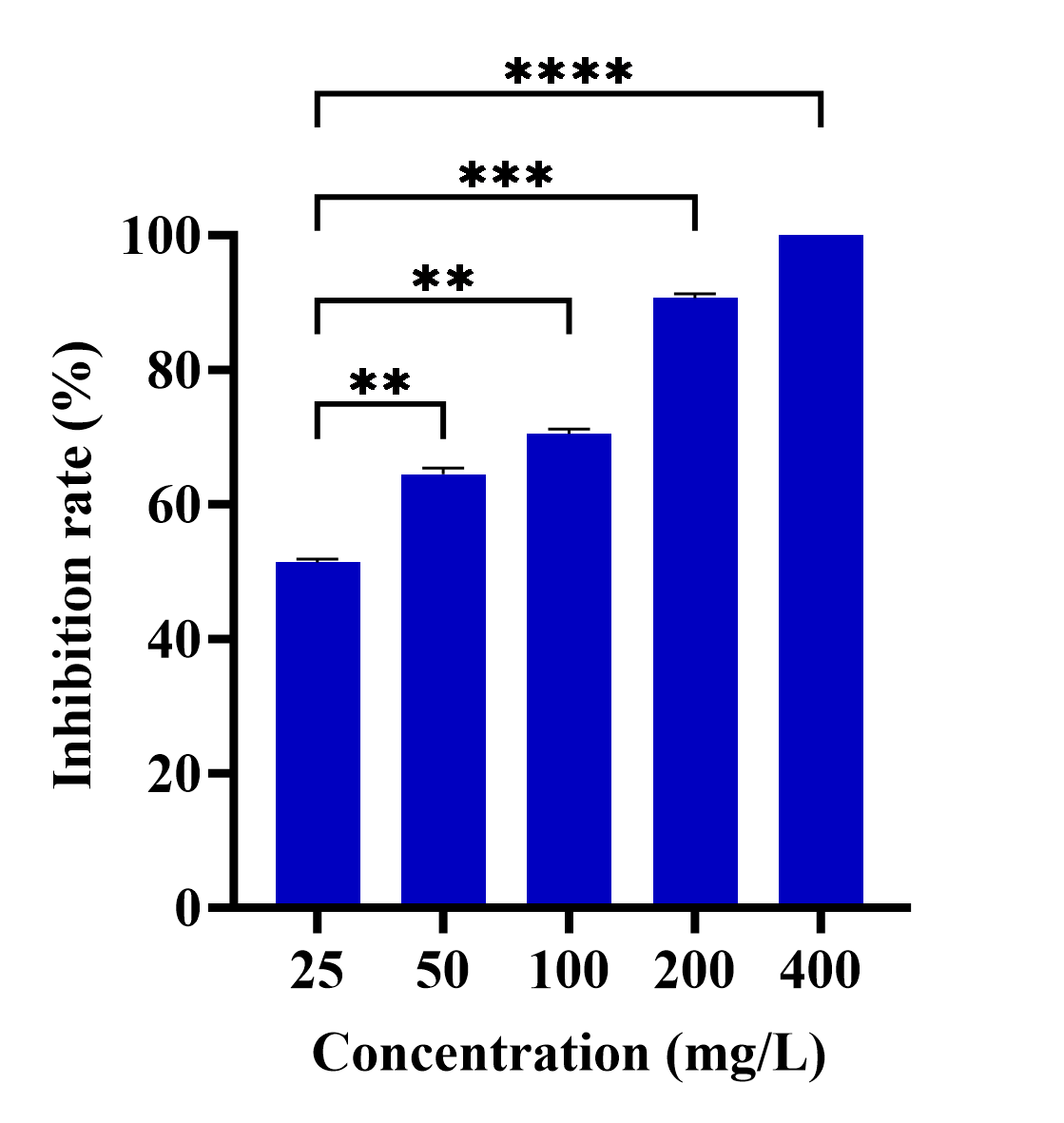
**

**Figure S15.** In vitro antibacterial activities of MOS@HPCD against *Psa* at 25, 50, 100, 200, and 400 mg/L after 48 h. *n* = 3. Results are shown as the mean ± SD. The statistical analysis was performed using a one-way analysis of variance (ANOVA). **p* < 0.05, ***p* < 0.01, ****p* < 0.001, *****p* < 0.0001.

Supplementary curative activity


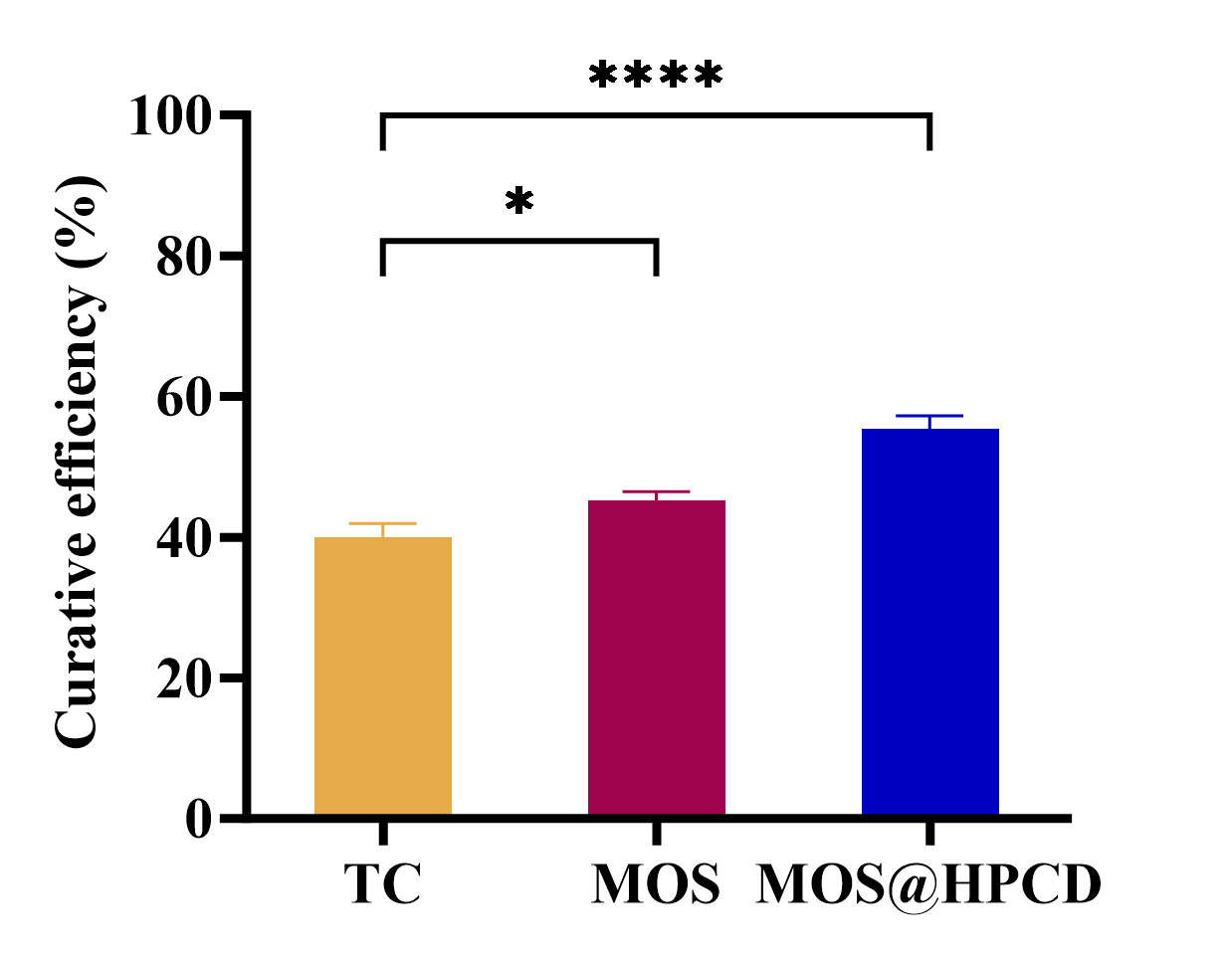


**Figure S16.** Curative activities of TC, MOS, and MOS@HPCD on *Psa*-induced kiwifruit leaf spots. TC, MOS, and MOS@HPCD were applied at a concentration of 500 g/ha. Data presented as mean ± SEM. Statistical analyses were performed using one-way ANOVA. **p* < 0.05, **** *p* < 0.0001. Yellow: TC, Red: MOS, Blue: MOS@HPCD.

Supplementary *Psa* abundances


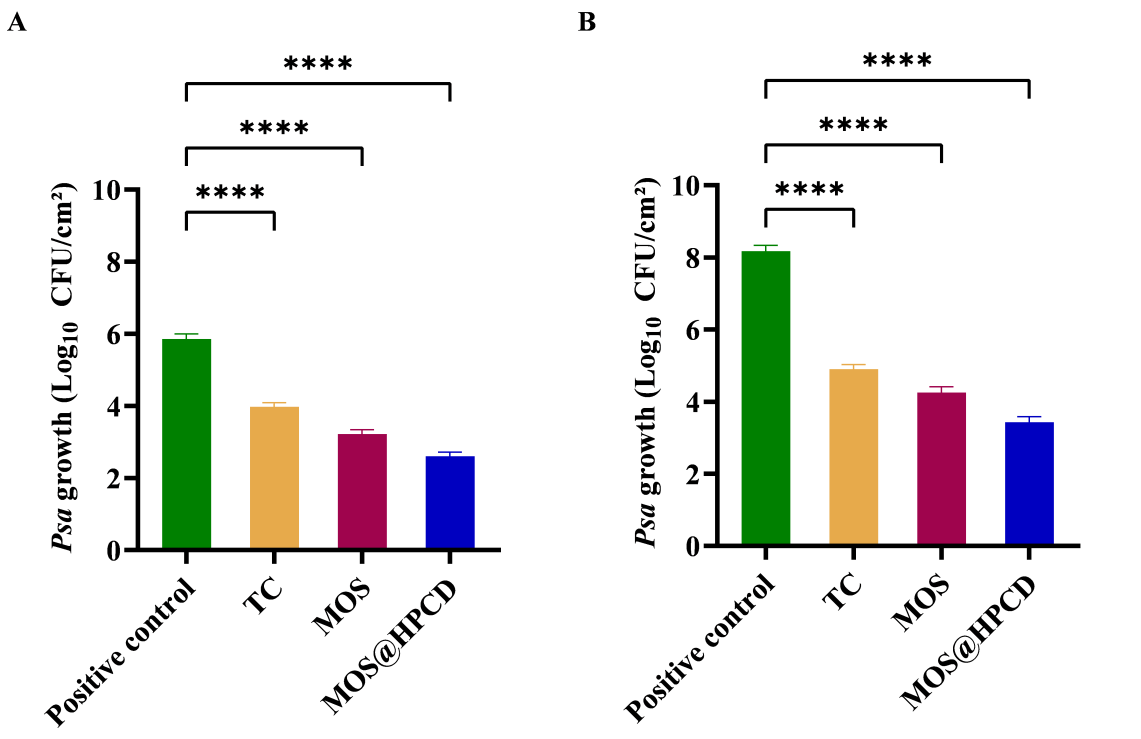


**Figure S17.** Effects of distilled water and 500 g/ha of each compound (TC, MOS, and MOS@HPCD) on the abundances of *Psa* in infected leaf discs. A) Protective and B) curative assays. Kiwifruit leaves infected by *Psa* and treated with distilled water were served as the positive control. Data are presented as mean ± SEM. Statistical analyses were performed using one-way ANOVA. **** *p* < 0.0001. Green: Positive control, Yellow: TC, Red: MOS, Blue: MOS@HPCD.

Supplementary kiwifruit security images


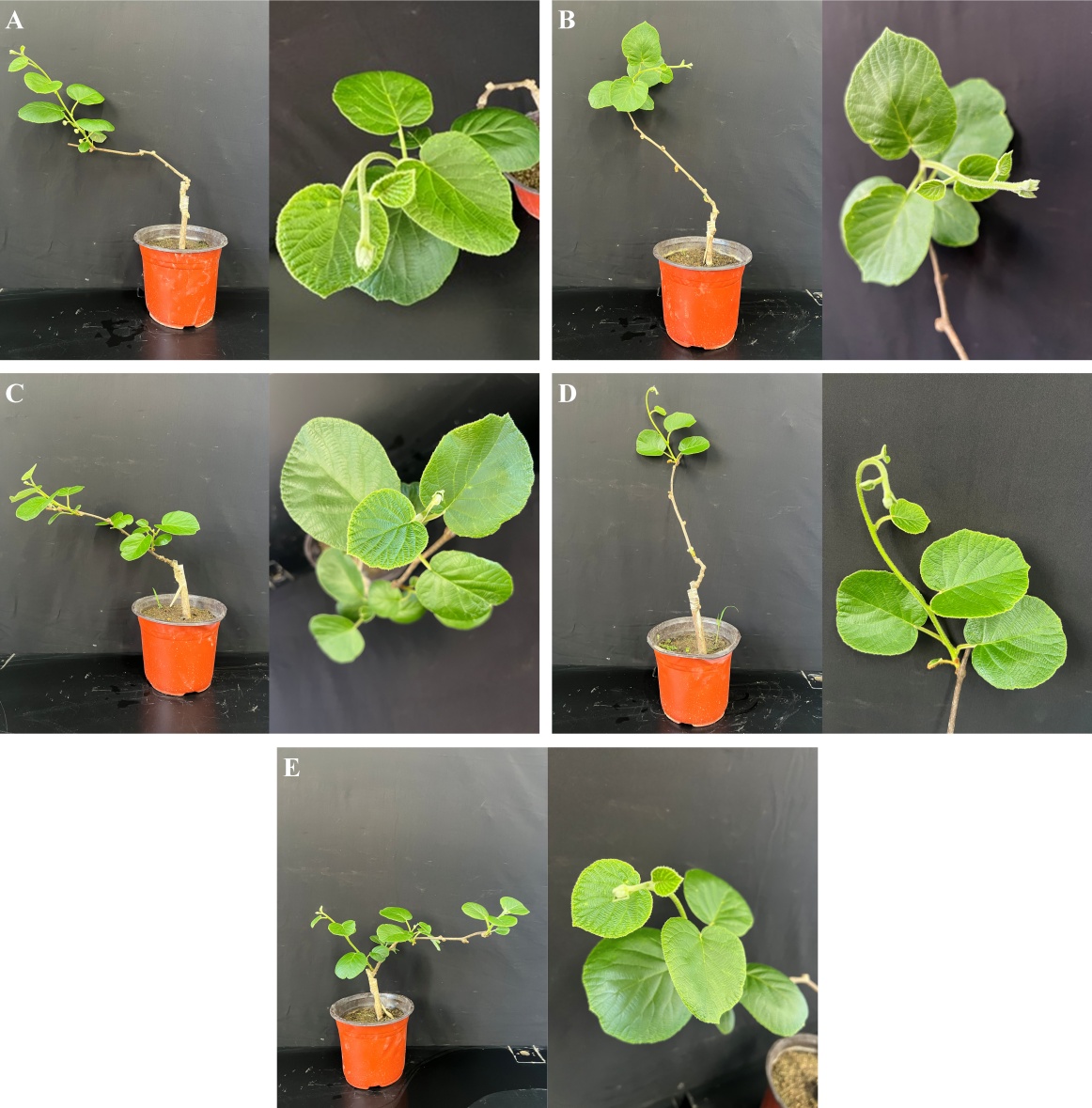


**Figure S18.** Security assessment of kiwifruits after 21-day treatment with CK, MOS, and MOS@HPCD at 500 and 1000 g/ha, respectively. (A) CK; (B) MOS, 500 g/ha; (C) MOS@HPCD, 500 g/ha; (D) MOS, 1000 g/ha; (E) MOS@HPCD, 1000 g/ha. Kiwifruit treated with distilled water only was used for the control check (CK).

Supplementary acute toxicity on zebrafish embryos at 96 h


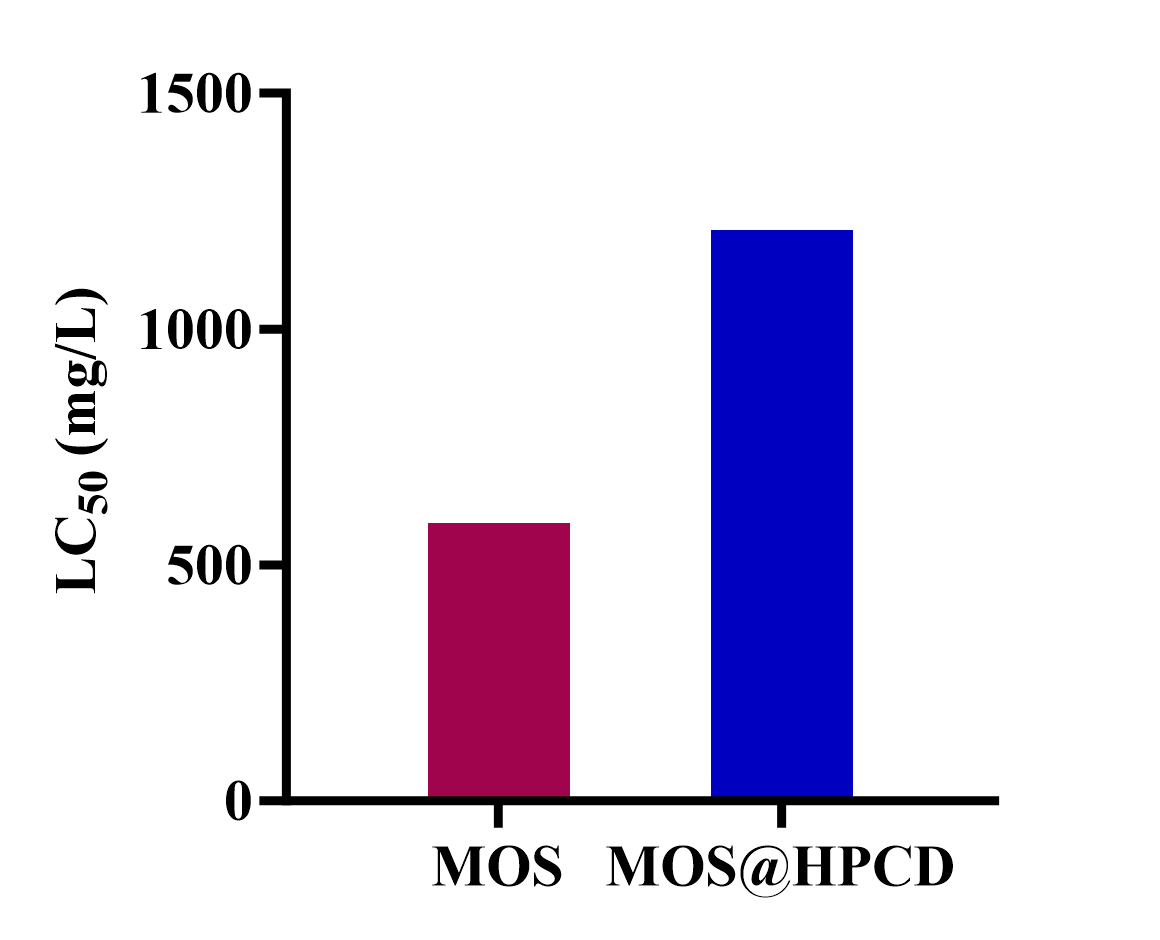


**Figure S19.** The acute toxicities of MOS, and MOS@HPCD toward zebrafish embryos at 96 h. Red: MOS, blue: MOS@HPCD.

Supplementary cytotoxicity evaluation

**Figure S20.** The cytotoxicity of MOS and MOS@HPCD toward human skin fibroblasts (HSF) cells after 24 h. The half maximal inhibitory concentration (IC_50_) values were calculated by probit regression modeling. Red: MOS, blue: MOS@HPCD.

Supplementary pharmacokinetic experiments in rats


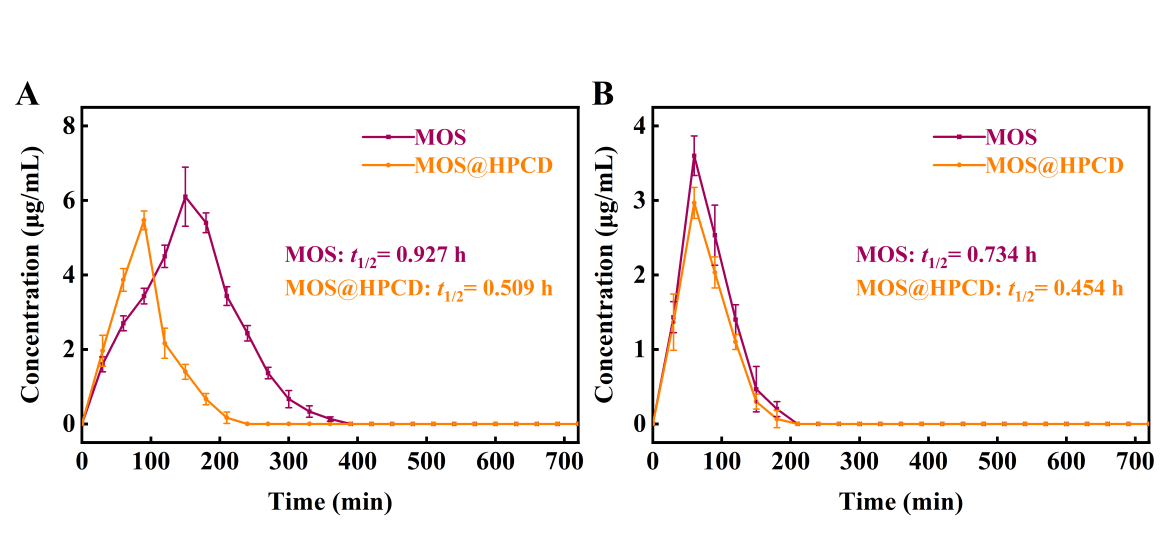


**Figure S21.** The pharmacokinetic curves of MOS and MOS@HPCD in rats over a period of 24 h. *n* = 3. Error bars, SD of three replications. Red: MOS, yellow: MOS@HPCD.

Supplementary crystal data and structure refinement

**Table S1.** Crystal data and structural refinement for MOS.

| **Compound** | **MOS** |
| --- | --- |
| Empirical formula | C_23_H_34_N_2_O_6_ |
| Formula weight | 434.52 |
| Temperature/K | 169.99(10) |
| Crystal system | orthorhombic |
| Space group | P2_1_2_1_2_1_ |
| a/Å | 9.5156(2) |
| b/Å | 13.4691(4) |
| c/Å | 17.1637(5) |
| α/° | 90 |
| β/° | 90 |
| γ/° | 90 |
| Volume/Å^3^ | 2,199.81(10) |
| Z | 4 |
| ρ_calc_g/cm^3^ | 1.312 |
| μ/mm^‑1^ | 0.775 |
| F(000) | 936.0 |
| Crystal size/mm^3^ | 0.15 × 0.1 × 0.08 |
| Radiation | Cu Kα (λ = 1.54184) |
| 2θ range for data collection/° | 8.344 to 147.192 |
| Index ranges | -11 ≤ h ≤ 10, -16 ≤ k ≤ 16, -9 ≤ l ≤ 21 |
| Reflections collected | 6016 |
| Independent reflections | 3839 [R_int_ = 0.0303, R_sigma_ = 0.0462] |
| Data/restraints/parameters | 3,839/0/285 |
| Goodness-of-fit on F^2^ | 1.019 |
| Final R indices [I>=2σ (I)] | R_1_ = 0.0484, wR_2_ = 0.1277 |
| Final R indices [all data] | R_1_ = 0.0531, wR_2_ = 0.1351 |
| Largest diff. peak/hole / e Å^-3^ | 0.20/-0.28 |
| Flack parameter | -0.04(19) |

Supplementary elemental analysis

**Table S2.** Elemental analysis of MOS.

| **Compound** | **Theoretical value (calculated value) (wt%)** | | |
| --- | --- | --- | --- |
|  | **C** | **H** | **N** |
| MOS | 66.3 (66.2) | 7.7 (7.7) | 6.7 (6.6) |

Supplementary hydrogen bonds of MOS

**Table S3.** Hydrogen bonds of MOS.

| **D** | **H** | **A** | **d(D-H)/Å** | **d(H-A)/Å** | **d(D-A)/Å** | **D-H-A/°** |
| --- | --- | --- | --- | --- | --- | --- |
| O4 | H4 | O3 | 0.82 | 1.81 | 2.535 (3) | 147.2 |
| N1 | H1 | O2^a^ | 0.98 | 1.78 | 2.740 (3) | 165.8 |

^a^1/2-X,1-Y,-1/2+Z.

Supplementary docker energies

**Table S4.** The docker energies of 5-OMESA, MT, and MOS with FtsZ and DNA helicase in *Psa*.

| **Compound** | **Docker energy (kcal/mol)** | |
| --- | --- | --- |
|  | **FtsZ** | **DNA helicase** |
| 5-OMESA | -5.619 | -5.000 |
| MT | -6.043 | -4.831 |
| MOS | -6.692 | -6.769 |

Supplementary stability analysis

**Table S5.** The particle sizes and pH values of MOS@HPCD at 0, 25, and 45 °C at 1, 2, 3, 4, 5, 6, and 7 days, respectively.

| **Time**  **(d)** | **4 °C** | | **25 °C** | | **45 °C** | |
| --- | --- | --- | --- | --- | --- | --- |
|  | **Particle size (nm)** | **pH** | **Particle size (nm)** | **pH** | **Particle size (nm)** | **pH** |
| 0 | - | - | 409 ± 3 | 6.62 ± 0.04 | - | - |
| 1 | 402 ± 4 | 7.12 ± 0.03 | 402 ± 4 | 6.61 ± 0.03 | 412 ± 3 | 6.69 ± 0.04 |
| 2 | 408 ± 4 | 6.62 ± 0.03 | 413 ± 3 | 6.66 ± 0.03 | 406 ± 5 | 6.74 ± 0.05 |
| 3 | 412 ± 5 | 6.63 ± 0.04 | 415 ± 6 | 6.64 ± 0.03 | 419 ± 4 | 6.82 ± 0.04 |
| 4 | 406 ± 6 | 6.66 ± 0.02 | 409 ± 2 | 6.65 ± 0.02 | 408 ± 3 | 6.94 ± 0.03 |
| 5 | 402 ± 5 | 6.64 ± 0.05 | 417 ± 7 | 6.65 ± 0.02 | 411 ± 4 | 7.00 ± 0.04 |
| 6 | 413 ± 6 | 6.63 ± 0.03 | 416 ± 6 | 6.65 ± 0.03 | 415 ± 3 | 7.12 ± 0.03 |
| 7 | 413 ± 3 | 6.68 ± 0.06 | 415 ± 2 | 6.61 ± 0.05 | 419 ± 6 | 7.20 ± 0.04 |

*n* = 3. Results are shown as the mean ± SD.

Supplementary evaluation of safety in kiwifruit

**Table S6.** Phytotoxicity assessment of kiwifruits after 21-day treatment with CK, MOS, and MOS@HPCD at 500 and 1000 g/ha, respectively.

| **Treatment** | **Dosage (g/ha)** | **Phytotoxicity**  **classification** | **Average number**  **of leaves**  **(0 d)** | **Average number of leaves**  **(21 d)** |
| --- | --- | --- | --- | --- |
| CK | 0 | - | 3.0 ± 0.0 | 6.0 ± 0.0 |
| MOS | 500 | - | 3.0 ± 0.0 | 6.0 ± 0.0 |
|  | 1000 | - | 3.0 ± 0.0 | 6.0 ± 0.0 |
| MOS@HPCD | 500 | - | 3.0 ± 0.0 | 6.0 ± 0.0 |
|  | 1000 | - | 3.0 ± 0.0 | 6.5 ± 0.6 |

"–" indicates no observed phytotoxicity. Kiwifruit treated with distilled water only was used for the control check (CK). *n* = 4. Results are shown as the mean ± SD.

Supplementary acute toxicity toward mice

**Table S7.** The acute toxicities of MOS and MOS@HPCD toward mice after 14 d.

| **Treatment** | **LD_50_ (mg/kg)** | **95% Confidence interval** | **Toxicity regression equation** | **R** |
| --- | --- | --- | --- | --- |
| MOS | 513.9 | 461.77-572.00 | y=-20.7693+9.5058x | 0.9852 |
| MOS@HPCD | MTD＞1,000 | - | - | - |

LD_50_: median lethal dose; MTD: maximal tolerated dose.

Supplementary transmembrane process of 5-OMESA

**Video S1.** The molecular dynamic trajectory of the membrane penetration of 5-OMESA.

Supplementary transmembrane process of MT

**Video S2.** The molecular dynamic trajectory of the membrane penetration of MT.

Supplementary transmembrane process of MOS

**Video S3.** The molecular dynamic trajectory of the membrane penetration of MOS.

References

1. H. Wang, Q. Mi, Y. Mao, Y. Tan, M. Yang, W. Liu, N. Wang, X. Tian, L. Huang, *J. Agric. Food. Chem.* **2024**, *72*, 2624-2633.
